# Sex Differences in Genetic Associations With Longevity

**DOI:** 10.1001/jamanetworkopen.2018.1670

**Published:** 2018-08-24

**Authors:** Yi Zeng, Chao Nie, Junxia Min, Huashuai Chen, Xiaomin Liu, Rui Ye, Zhihua Chen, Chen Bai, Enjun Xie, Zhaoxue Yin, Yuebin Lv, Jiehua Lu, Jianxin Li, Ting Ni, Lars Bolund, Kenneth C. Land, Anatoliy Yashin, Angela M. O’Rand, Liang Sun, Ze Yang, Wei Tao, Anastasia Gurinovich, Claudio Franceschi, Jichun Xie, Jun Gu, Yong Hou, Xiao Liu, Xun Xu, Jean-Marie Robine, Joris Deelen, Paola Sebastiani, Eline Slagboom, Thomas Perls, Elizabeth Hauser, William Gottschalk, Qihua Tan, Kaare Christensen, Xiaoming Shi, Mike Lutz, Xiao-Li Tian, Huanming Yang, James Vaupel

**Affiliations:** 1 Center for the Study of Aging and Human Development, Medical School of Duke University, Durham, North Carolina; 2 Center for Healthy Aging and Development Studies, National School of Development, Raissun Institute for Advanced Studies, Peking University, Beijing, China; 3 BGI Education Center, University of Chinese Academy of Sciences, Shenzhen, China; 4 BGI–Shenzhen, Shenzhen, China; 5 The First Affiliated Hospital, Institute of Translational Medicine, School of Medicine, Zhejiang University, Hangzhou, China; 6 Business School of Xiangtan University, Xiangtan, China; 7 Division of Non-Communicable Disease Control and Community Health, Chinese Center for Disease Control and Prevention, Beijing, China; 8 National Institute of Environmental Health, Chinese Center for Disease Control and Prevention, Beijing, China; 9 Department of Sociology, Peking University, Beijing, China; 10 School of Life Sciences, Fudan University, Shanghai, China; 11 Department of Biomedicine, Aarhus University, Aarhus, Denmark; 12 Duke Population Research Institute, Duke University, Durham, North Carolina; 13 The MOH Key Laboratory of Geriatrics, Beijing Hospital, National Center of Gerontology, Beijing, China; 14 School of Life Sciences, Peking University, Beijing, China; 15 Boston University, Boston, Massachusetts; 16 University of Bologna, Bologna, Italy; 17 Department of Biostatistics and Bioinformatics, Duke University, Durham, North Carolina; 18 French National Institute on Health and Medical Research and Ecole Pratique des Hautes Etudes, University of Montpellier, Montpellier, France; 19 Max Planck Institute for Biology of Ageing, Cologne, Germany; 20 Department of Molecular Epidemiology, Leiden University Medical Center, Leiden, the Netherlands; 21 Molecular Physiology Institute, Medical Center, Duke University, Durham, North Carolina; 22 Department of Neurology, Medical Center, Duke University, Durham, North Carolina; 23 University of Southern Denmark, Odense, Denmark; 24 Human Aging Research Institute and School of Life Science, Nanchang University, Jiangxi, China; 25 James D. Watson Institute of Genome Sciences, Hangzhou, China; 26 Max Planck Institute for Demographic Research, Rostock, Germany

## Abstract

**Importance:**

Sex differences in genetic associations with human longevity remain largely unknown; investigations on this topic are important for individualized health care.

**Objective:**

To explore sex differences in genetic associations with longevity.

**Design, Setting, and Participants:**

This population-based case-control study used sex-specific genome-wide association study and polygenic risk score (PRS) analyses to examine sex differences in genetic associations with longevity. Five hundred sixty-four male and 1614 female participants older than 100 years were compared with a control group of 773 male and 1526 female individuals aged 40 to 64 years. All were Chinese Longitudinal Healthy Longevity Study participants with Han ethnicity who were recruited in 1998 and 2008 to 2014.

**Main Outcomes and Measures:**

Sex-specific loci and pathways associated with longevity and PRS measures of joint effects of sex-specific loci.

**Results:**

Eleven male-specific and 11 female-specific longevity loci (*P* < 10^−5^) and 35 male-specific and 25 female-specific longevity loci (10^−5^ ≤ *P* < 10^−4^) were identified. Each of these loci’s associations with longevity were replicated in north and south regions of China in one sex but were not significant in the other sex (*P* = .13-.97), and loci-sex interaction effects were significant (*P* < .05). The associations of loci rs60210535 of the *LINC00871* gene with longevity were replicated in Chinese women (*P* = 9.0 × 10^−5^) and US women (*P* = 4.6 × 10^−5^) but not significant in Chinese and US men. The associations of the loci rs2622624 of the *ABCG2* gene were replicated in Chinese women (*P* = 6.8 × 10^−5^) and European women (*P* = .003) but not significant in both Chinese and European men. Eleven male-specific pathways (inflammation and immunity genes) and 34 female-specific pathways (tryptophan metabolism and PGC-1α induced) were significantly associated with longevity (*P* < .005; false discovery rate < 0.05). The PRS analyses demonstrated that sex-specific associations with longevity of the 4 exclusive groups of 11 male-specific and 11 female-specific loci (*P* < 10^−5^) and 35 male-specific and 25 female-specific loci (10^−5^ ≤*P* < 10^−4^) were jointly replicated across north and south discovery and target samples. Analyses using the combined data set of north and south showed that these 4 groups of sex-specific loci were jointly and significantly associated with longevity in one sex (*P* = 2.9 × 10^−70^ to 1.3 × 10^−39^) but not jointly significant in the other sex (*P* = .11 to .70), while interaction effects between PRS and sex were significant (*P* = 4.8 × 10^−50^ to 1.2 × 10^−16^).

**Conclusion and Relevance:**

The sex differences in genetic associations with longevity are remarkable, but have been overlooked by previously published genome-wide association studies on longevity. This study contributes to filling this research gap and provides a scientific basis for further investigating effects of sex-specific genetic variants and their interactions with environment on healthy aging, which may substantially contribute to more effective and targeted individualized health care for male and female elderly individuals.

## Key Points

**Question:**

Are there sex differences in genetic associations with longevity?

**Findings:**

In this case-control study of 2178 cases and 2299 controls who were Chinese with Han ethnicity, sex-specific genome-wide association study and sex-specific polygenic risk score analyses on longevity showed substantial and significant differences in genetic associations with longevity between men and women. Findings indicated that previously published genome-wide association studies on longevity identified some sex-independent genetic variants but missed sex-specific longevity loci and pathways.

**Meaning:**

These novel findings contribute to filling the gaps in the research literature, and further investigations may substantially contribute to individualized health care and more effective and targeted health interventions for male and female elderly individuals.

## Teaser

This population-based case-control study of Chinese Longitudinal Healthy Longevity Study participants uses sex-specific genome-wide association study (GWAS) and polygenic risk score (PRS) analyses to examine sex differences in genetic associations with longevity by comparing participants older than 100 years with a control group of participants aged 40 to 64 years.

## Introduction

Centenarian genomes may harbor genetic variants associated with longevity and health,^1,2,3,4,5^ supported by the fact that the proportion of genetic variants positively (or negatively) associated with longevity and health is significantly higher (or lower) among centenarians compared with middle-aged controls. This is because those who carry the longevity-favoring genetic variants have a better chance of surviving to age 100 years or older, while those with less favorable genetic variants may not reach 100 years. This relationship has been demonstrated empirically^1,2,3,4,5,6^ and proven mathematically.^6^ Hence, all of the genome-wide association studies (GWAS) on longevity use centenarians (and/or those aged ≥90 years or ≥85 years) as cases and younger adults as controls^2,3,4,5^ (eAppendix section S1 in the Supplement).

The extant literature indicates that associations of some genetic variants with health outcomes differ significantly between men and women.^7,8,9^ A recent study using the phenotype of parental age at death as an outcome variable indicated that different genes may be associated with longevity in men and women.^10^ However, sex differences have been overlooked in all previously published GWAS on longevity that used male and female combined data sets adjusted for sex as a covariate.^2,3,4,5^ A few GWAS of longevity conducted sex-specific analyses on the significant loci that were replicated in the combined male and female discovery and evaluation stages, but none of those studies found that their replicated loci had significant sex differences in the association with longevity.^2,3,4,5^ This is because, statistically, if the tested variable is significant in one sex but not significant in the other sex, it cannot be significant and replicated in the combined data sets, as the results of 2 sexes offset each other in a combined data set of male and female results, while the sample size of either one of the sexes is usually not small enough to leave the overall results unaffected.^11^ In other words, all previously published GWAS on longevity identified sex-independent genetic variants, but the sex differences have been overlooked. The present study aims to fill this research gap and contribute to a better understanding of sex differences in genetic associations with longevity.

## Methods

We analyzed Chinese Longitudinal Healthy Longevity Study (CLHLS) data sets of GWAS on longevity, with 564 male and 1614 female participants aged 100 years or older (mean [SD] age, 102.7 [3.49] years) as cases and 773 male and 1526 female participants aged 40 to 64 years (mean [SD] age, 48.4 [7.44] years) as controls. All were Chinese with Han ethnicity (eAppendix sections S2-S3 in the Supplement). The CLHLS GWAS has the largest sample size of centenarians in the world, 2.7 times as large as the next largest sample of centenarians of GWAS on longevity. The CLHLS GWAS includes 5.6 million single-nucleotide polymorphisms (SNPs) (0.82 million genotyped SNPs and 4.8 million imputed SNPs) for each of the centenarians and middle-aged controls (eAppendix section S3.1 in the Supplement).^5^ The CLHLS GWAS followed the Strengthening the Reporting of Genetic Association Studies (STREGA) reporting guideline for GWAS quality control,^12^ including genotyping errors, population stratification, and Hardy-Weinberg equilibrium, with a full quality item score of 12, indicating good quality and completeness.^5^ The Research Ethics Committees of Peking University and Duke University granted approval for the Protection of Human Subjects for the CLHLS, including collections of questionnaire data and DNA samples with written informed consent before participation.

The Chinese with Han ethnicity make up about 93% of the total population in China, with 53 Chinese minority groups making up 7% of the total population. The sample sizes of any minority group in the CLHLS data are too small for meaningful analysis, so we included Han Chinese samples only in the present study.^5^ Detailed descriptions of the CLHLS phenotype and genotype data sets are presented in eAppendix sections S2 and S3 in the Supplement.

We adopted a stratification framework of north and south regions of China as discovery and evaluation samples (eAppendix section S3.2, eTable 1, and eFigures 1-4 in the Supplement), following most published case-control genetic studies using Chinese nationwide data sets and based on analyses of principal components, genetics (classic markers, microsatellite DNA markers, mitochondrial DNA, and Y chromosome SNP markers), anthropology, and linguistics, reported in the literature.^13^

We conducted 2-stage consecutive analyses, with sex-specific GWAS to identify candidate sex-specific loci and sex-specific pathways in stage 1 and polygenic risk score (PRS) analysis in stage 2 (Figure). To avoid the high false-negative rate and to fully use the available independent GWAS data sets of north and south regions of China, we applied the bidirectional discovery and evaluation approach^14^ (eAppendix section S4 in the Supplement) in our sex-specific GWAS and PRS analyses. A priori thresholds of *P* < 10^−5^, *P* < 10^−4^, or *P* < 10^−3^ or higher were defined for selecting informative SNPs in the discovery step of recent GWAS or PRS studies depending on the circumstances of the research, while *P* < 5 × 10^−8^ is the standard for genome-wide significance.^15^ We aimed to identify groups of sex-specific SNPs that individually may have very small effects but may jointly have large effects. Thus, it is reasonable to choose a modest a priori threshold of *P* < 10^−3^ and *P* < .01 in the discovery step of sex-specific single SNP analysis. We performed sex-specific GWAS using PLINK (version 1.06).^16^ To minimize population stratification effects, we adjusted for the top 2 eigenvectors, which corrected nearly all of the stratification that can be corrected.^17^ In the combined north and south data analysis, we also adjusted for respective north and south regions.

**Figure.**
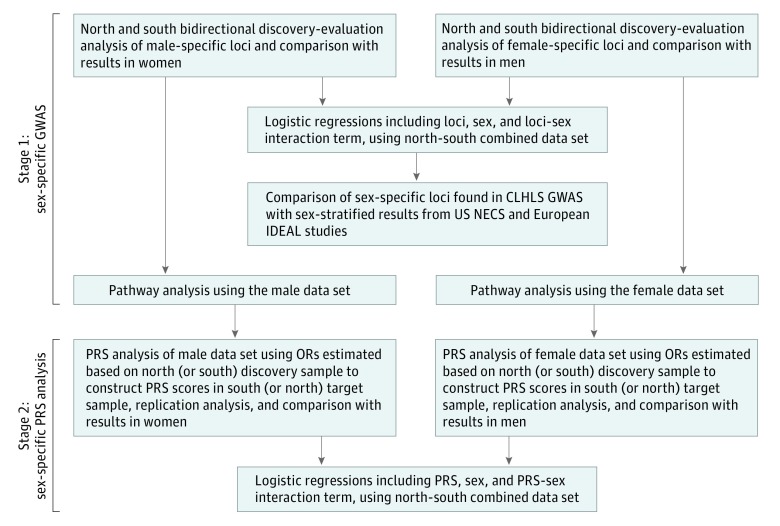
Flowchart of the 2-Stage Consecutive Analyses Stage 1 was a sex-specific genome-wide association study (GWAS) that analyzed single-nucleotide polymorphisms (SNPs) and pathways. Stage 2 was a sex-specific polygenic risk score (PRS) analysis. CLHLS indicates Chinese Longitudinal Healthy Longevity Study; IDEAL, European Union Longevity Genetics Consortium; NECS, New England Centenarians Study; OR, odds ratio.

The best-fit *P* value cutoffs .0042 and .02 (calculated by PRSice software with the BEST_FIT command^18^) were used to select SNPs for pathway analyses in men and women, respectively. We implemented an improved gene set enrichment analysis for GWAS using the i-GSEA4GWAS database^19^ to map genes to pathways. Sex-specific pathway gene sets with *P* < .005 and false discovery rate (FDR) < 0.05 were regarded as significantly associated with longevity.

We conducted PRS analyses in stage 2 based on 2 considerations. First, each of the candidate sex-specific loci identified in stage 1 had a very small effect, leading to further assessment of their joint effects by PRS analyses. Second, the candidate sex-specific loci selected in stage 1 were individually not significant (*P* > .05) in the other sex, but their joint effects could be large and significant in the other sex (eAppendix section S5 in the Supplement); PRS analyses allowed us to evaluate and filter out those loci that are not truly sex specific.

Using PRSice software^18^ and standard methods,^20^ we constructed PRS scores as the sum of the number of risk allele copies of each of the selected loci multiplied by the log of the corresponding odds ratio of longevity, and then divided by the total number of selected loci for each of the centenarians and middle-aged controls. We conducted analysis including a PRS-sex interaction term based on the continuous PRS. We used the PRSice clumping method to select independent loci by excluding all SNPs with linkage disequilibrium (*r*^2^ > 0.1); only independent loci were used to calculate the PRS.

Following standard procedures,^20^ we used the sex-specific odds ratios estimated based on the discovery sample of north (or south) region as weights to construct the PRS in the target sample of south (or north) region; we also conducted the PRS analysis on the sex-specific loci that were replicated across discovery and target samples, using the north-south combined data set.

## Results

### Analyses of Single SNPs 

Results in Table 1 indicate 11 independent male-specific loci (including the SNP rs1950902 in the *MTHFD1* gene) associated with longevity that replicate in the male discovery and evaluation data sets of north and south regions (with *P* < 10^−3^ in the discovery step) and reached *P* < 10^−5^ and FDR < 10^−4^ in the male north-south combined data set, but were not significant (*P* = .17-.95) in the female north-south combined data set. The loci-sex interaction effects of these loci were significant (*P* = 8.40 × 10^−6^ to 8.45 × 10^−4^).

**Table 1.** The 11 Male-Specific Loci Associated With Longevity and Replicated in North and South Data Sets^a^

Loci	Chromosome	Nearby Gene	North and South Discovery-Evaluation Analysis of Male-Specific Loci								Female Data Set (North and South Combined)			*P* Value of Loci-Sex Interaction Effects^c^	Regression Adjusted for Sex as a Covariate Without Loci-Sex Interaction Term, Using Combined Data Set	
			North^b^		South		Combined North and South									
			*P *Value	OR (95% CI)	*P *Value	OR (95% CI)	MAF (Case vs Control)	*P *Value	FDR	OR (95% CI)	MAF (Case vs Control)	*P *Value	OR (95% CI)		*P *Value	OR (95% CI)
rs1950902	14	*MTHFD1*	5.0 × 10^-4^	1.515 (1.23-1.90)	1.4 × 10^-4^	1.649 (1.24-2.20)	0.37 vs 0.26	1.1 × 10^-7^	1.4 × 10^-6^	1.595 (1.34-1.90)	0.32 vs 0.31	.95	1.004 (0.90-1.12)	8.4 × 10^-6^	3.6 × 10^-3^	1.145 (1.05-1.26)
rs1157755	12	*KCNA5*	3.0 × 10^-4^	2.565 (1.28-3.29)	1.3 × 10^-3^	2.446 (1.88-7.15)	0.08 vs 0.03	1.9 × 10^-6^	7.9 × 10^-6^	2.468 (1.70-3.58)	0.06 vs 0.06	.38	0.907 (0.73-1.13)	5.5 × 10^-6^	6.9 × 10^-2^	1.188 (0.98-1.42)
rs11136774	8	*CSMD1*	1.8 × 10^-4^	1.600 (1.17-1.88)	7.6 × 10^-3^	1.475 (1.14-2.13)	0.30 vs 0.21	2.6 × 10^-6^	8.0 × 10^-6^	1.560 (1.30-1.88)	0.25 vs 0.25	.85	0.988 (0.88-1.11)	5.0 × 10^-5^	1.6 × 10^-2^	1.131 (1.02-1.25)
rs6453914	6	*IMPG1*	9.1 × 10^-4^	1.581 (1.23-2.07)	2.9 × 10^-3^	1.633 (1.11-2.26)	0.23 vs 0.16	4.1 × 10^-6^	9.7 × 10^-6^	1.624 (1.32-2.00)	0.19 vs 0.18	.40	1.057 (0.93-1.20)	5.7 × 10^-4^	1.6 × 10^-3^	1.192 (1.07-1.33)
rs6740706	2	*LRRFIP1*	9.4 × 10^-7^	0.536 (0.48-0.77)	3.9 × 10^-2^	0.734 (0.47-0.90)	0.20 vs 0.29	2.3 × 10^-7^	1.2 × 10^-5^	0.610 (0.51-0.74)	0.23 vs 0.25	.39	0.950 (0.85-1.07)	2.9 × 10^-4^	3.6 × 10^-4^	0.837 (0.76-0.92)
rs12199884	6	*PKHD1*	9.8 × 10^-4^	0.365 (0.20-0.60)	9.5 × 10^-4^	0.464 (0.29-0.79)	0.04 vs 0.08	4.1 × 10^-6^	8.1 × 10^-6^	0.428 (0.30-0.61)	0.06 vs 0.06	.94	0.992 (0.80-1.24)	9.8 × 10^-5^	4.2 × 10^-3^	0.767 (0.65-0.93)
rs79072042	5	*NUDT12*	3.7 × 10^-5^	0.470 (0.38-0.74)	4.9 × 10^-2^	0.678 (0.39-0.93)	0.09 vs 0.15	7.2 × 10^-6^	1.5 × 10^-6^	0.552 (0.43-0.72)	0.13 vs 0.13	.60	0.961 (0.83-1.12)	8.3 × 10^-5^	5.8 × 10^-3^	0.837 (0.73-0.94)
rs200536623	1	*SYDE2*	7.4 × 10^-5^	5.917 (2.29-12.06)	1.5 × 10^-2^	3.553 (1.22-11.6)	0.04 vs 0.01	8.8 × 10^-6^	1.2 × 10^-5^	4.527 (2.33-8.81)	0.02 vs 0.02	.54	1.136 (0.75-1.71)	5.5 × 10^-4^	1.8 × 10^-3^	1.726 (1.25-2.48)
rs138863	22	*BRD1*	6.9 × 10^-3^	0.270 (0.11-0.66)	5.4 × 10^-4^	0.200 (0.07-0.50)	0.01 vs 0.04	9.5 × 10^-6^	1.1 × 10^-5^	0.220 (0.12-0.44)	0.02 vs 0.03	.17	0.790 (0.57-1.10)	8.5 × 10^-4^	1.3 × 10-4	0.575 (0.44-0.77)
rs9894443	17	*SLC39A11*	2.6 × 10^-3^	1.390 (1.13-1.70)	6.3 × 10^-4^	1.560 (1.25-2.19)	0.42 vs 0.34	8.2 × 10^-6^	1.2 × 10^-5^	1.450 (1.23-1.70)	0.35 vs 0.37	.41	0.960 (0.86-1.06)	2.6 × 10^-5^	8.8 × 10^-2^	1.078 (0.99-1.18)
rs73329622	5	*STK10*	3.3 × 10^-3^	0.680 (0.57-0.93)	9.5 × 10^-4^	0.600 (0.39-0.75)	0.18 vs 0.26	9.2 × 10^-6^	1.0 × 10^-5^	0.640 (0.53-0.78)	0.22 vs 0.22	.88	0.990 (0.88-1.12)	2.4 × 10^-4^	9.1 × 10^-3^	0.872 (0.78-0.96)

Abbreviations: FDR: false discovery rate; MAF, minor allele frequency; OR, odds ratio.

^a^ As discussed in eAppendix section S2 in the Supplement, the ORs in this and other Tables cannot be interpreted as the size of pure effects of the genotype on longevity because they are estimated based on the differences of the proportions of carrying the genotype between the cases (centenarians) and controls (middle-aged adults) and these proportions also depend on other factors, such as gene-environment interaction effects.

^b^ The sex-specific subsample sizes of north, south, and north-south combined regions are listed in eTable 1 in the Supplement.

^c^ The estimates of *P* value of loci-sex interaction effects are based on logistic regressions including the loci, sex, and loci-sex interaction term, using the north-south combined data set.

As shown in Table 2, we identified 11 independent female-specific loci (including the SNP rs1027238 at the *FAM19A1* gene and the SNP rs2161877 near *TBX3*) whose associations with longevity were replicated in female discovery and evaluation data sets of north and south regions (with *P* < 10^−3^ in the discovery step) and reached *P* < 10^−5^ and FDR < 10^−4^ in the female north-south combined data set, but were not significant (*P* = .13-.97) in the male north-south combined data set. The loci-sex interaction effects of these female-specific loci were significant (*P* = 2.8 × 10^−4^ to 2.5 × 10^−2^).

**Table 2.** The 11 Female-Specific Loci Associated With Longevity And Replicated in North and South Data Sets^a^

Loci	Chromosome	Nearby Gene	North and South Discovery-Evaluation Analysis of Female-Specific Loci								Male Data Set (North and South Combined)			*P* Value of Loci-Sex Interaction Effects^c^	Regression Adjusted for Sex as a Covariate Without Loci-Sex Interaction Term, Using Combined Data Set	
			North^b^		South		Combined North and South									
			*P *Value	OR (95% CI)	*P *Value	OR (95% CI)	MAF (Case vs Control)	*P *Value	FDR	OR (95% CI)	MAF (Case vs Control)	*P *Value	OR (95% CI)		*P* Value	OR (95% CI)
rs12568089	1	*ZFP69B*	8.1 × 10^-4^	1.350 (1.17-1.64)	1.1 × 10^-3^	1.353 (1.06-1.56)	0.23 vs 0.17	2.7 × 10^-6^	3.1 × 10^-5^	1.352 (1.19-1.53)	0.22 vs 0.20	.81	1.024 (0.85-1.24)	.02	6.2 × 10^-5^	1.237 (1.11-1.37)
rs3805586	5	*PGGT1B*	1.2 × 10^-4^	1.342 (1.10-1.47)	2.4 × 10^-2^	1.196 (1.08-1.50)	0.35 vs 0.30	8.9 × 10^-6^	1.1 × 10^-5^	1.275 (1.15-1.42)	0.31 vs 0.33	.13	0.878 (0.74-1.04)	.0003	2.7 × 10^-3^	1.147 (1.05-1.26)
rs1027238	3	*FAM19A1*	4.6 × 10^-4^	0.636 (0.49-0.80)	1.8 × 10^-3^	0.667 (0.53-0.91)	0.07 vs 0.11	2.8 × 10^-6^	1.6 × 10^-5^	0.652 (0.55-0.78)	0.09 vs 0.08	.37	1.136 (0.86-1.50)	.001	5.1 × 10^-4^	0.766 (0.66-0.89)
rs12711357	4	*FSTL5*	1.3 × 10^-4^	0.736 (0.65-0.88)	.01	0.786 (0.61-0.92)	0.20 vs 0.25	9.1 × 10^-6^	1.0 × 10^-5^	0.763 (0.68-0.86)	0.23 vs 0.24	.75	0.970 (0.81-1.17)	.03	7.9 × 10^-5^	0.818 (0.74-0.90)
rs416352	6	*NOTCH4*	2.5 × 10^-3^	0.810 (0.73-0.94)	5.1 × 10^-4^	1.320 (1.18-1.65)	0.51 vs 0.47	7.8 × 10^-6^	1.5 × 10^-5^	1.261 (1.14-1.40)	0.51 vs 0.48	.93	0.993 (0.86-1.18)	.01	2.5 × 10^-4^	1.173 (1.08-1.28)
rs73070152	19	*KIR3DX1*	1.3 × 10^-3^	1.440 (1.17-1.81)	7.1 × 10^-4^	1.600 (1.20-2.15)	0.12 vs 0.08	8.0 × 10^-6^	1.3 × 10^-5^	1.477 (1.25-1.75)	0.10 vs 0.10	.97	1.004 (0.78-1.30)	.01	1.9 × 10^-4^	1.307 (1.14-1.50)
rs13406646	2	*CYP1B1*-*AS1*	6.6 × 10^-3^	1.280 (1.08-1.52)	5.0 × 10^-4^	1.430 (1.19-1.84)	0.21 vs 0.16	9.8 × 10^-6^	1.0 × 10^-5^	1.348 (1.18-1.54)	0.19 vs 0.19	.78	0.972 (0.80-1.19)	.007	6.1 × 10^-4^	1.210 (1.08-1.35)
rs2161877	12	*TBX3*	2.9 × 10^-3^	0.810 (0.69-0.90)	3.5 × 10^-4^	0.750 (0.64-0.90)	0.39 vs 0.46	2.7 × 10^-6^	1.0 × 10^-5^	0.778 (0.70-0.86)	0.41 vs 0.42	.72	0.971 (0.83-1.14)	.02	5.2 × 10^-5^	0.834 (0.76-0.91)
rs4972778	2	*KIAA1715*	1.5 × 10^-3^	0.780 (0.65-0.87)	8.4 × 10^-4^	0.720 (0.64-0.96)	0.20 vs 0.26	5.4 × 10^-6^	1.5 × 10^-5^	0.759 (0.67-0.85)	0.24 vs 0.25	.70	1.036 (0.87-1.24)	.004	3.0 × 10^-4^	0.834 (0.76-0.92)
rs118113034	6	*FRK*	2.8 × 10^-3^	0.410 (0.23-0.70)	6.9 × 10^-4^	0.180 (0.04-0.45)	0.01 vs 0.02	8.5 × 10^-6^	1.2 × 10^-5^	0.320 (0.19-0.53)	0.02 vs 0.02	.90	1.041 (0.57-1.91)	.003	2.0 × 10^-4^	0.487 (0.33-0.71)
rs12472681	2	*LOC1720*	1.3 × 10^-3^	1.910 (1.16-2.44)	5.6 × 10^-4^	2.280 (1.79-5.22)	0.05 vs 0.02	5.5 × 10^-6^	1.2 × 10^-5^	2.004 (1.49-2.70)	0.03 vs 0.04	.54	0.868 (0.55-1.37)	.003	3.3 × 10^-4^	1.557 (1.22-1.98)

Abbreviations: FDR: false discovery rate; MAF, minor allele frequency; OR, odds ratio.

^a^ As discussed in eAppendix section S2 in the Supplement, the ORs in this and other Tables cannot be interpreted as the size of pure effects of the genotype on longevity because they are estimated based on the differences of the proportions of carrying the genotype between the cases (centenarians) and controls (middle-aged adults) and these proportions also depend on other factors, such as gene-environment interaction effects.

^b^ The sex-specific subsample sizes of north, south, and north-south combined regions are listed eTable 1 in the Supplement.

^c^ The estimates of *P* value of loci-sex interaction effects are based on logistic regressions including the loci, sex, and loci-sex interaction term, using the north-south combined data set.

Following the widely practiced approach in the PRS literature,^18,20^ in addition to the 11 male-specific and 11 female-specific loci outlined, we also identified candidate sex-specific loci with a more relaxed prior threshold for further PRS analyses. With a prior threshold of *P* < .01 in the discovery step, we found that additional 47 male-specific and 34 female-specific independent loci were associated with longevity and replicated across north and south samples, had a 10^−5^ ≤ *P* < 10^−4^ in one sex but were not significant in the other sex, and had *P* < .05 for the loci-sex interaction effects, using the north-south combined data set. As discussed earlier, the 11 male-specific and 11 female-specific loci (*P* < 10^−5^) and 47 male-specific and 34 female-specific loci (10^−5^ ≤ *P* < 10^−4^) are individually candidates of sex-specific longevity loci, and whether their joint effects are truly sex-specific was investigated in the PRS analyses.

The Chinese sex-specific loci that were significant (*P* < 10^−4^) in one sex but not significant (*P* > .05) in the other sex and available in the New England Centenarians Study (NECS) and European Union Longevity Genetics Consortium (IDEAL) were tested for replication in NECS and IDEAL. The samples and data sources of GWAS on longevity from NECS and IDEAL are described by Sebastiani et al^2^ and Deelen et al.^3^ The results of comparisons across the Chinese CLHLS, the US NECS and European IDEAL presented in Table 3, show that rs60210535 of *LINC00871* replicated between Chinese (*P* = 9.0 × 10^−5^) and American (*P* = 4.6 × 10^−5^) women, but was not significant in both Chinese and American men (*P* = .49-.69). Another female-specific locus, rs2622624 of *ABCG2*, had *P* = 6.8 × 10^−5^ in Chinese women and *P* = .003 in European women but was not significant in both Chinese and European men (*P* = .08-.59). ABCG2 is a well-known breast cancer resistance protein (BCRP).^21^ *LINC00871* is a noncoding RNA gene, and its function is uncertain.

**Table 3.** Two Female-Specific Loci Associated With Longevity in the Han Chinese CLHLS Replicated in the US NECS or the European IDEAL

SNP	Chromosome	Position	Nearest Gene	Coded vs Noncoded Allele	Chinese CLHLS						US NECS^a^				European IDEAL^b^			
					Men			Women			Men		Women		Men		Women	
					MAF (Case vs Control)	*P *Value	OR (95% CI)	MAF (Case vs Control)	*P *Value	OR (95% CI)	*P *Value	OR (95% CI)	*P *Value	OR (95% CI)	*P *Value	Effect Direction	*P *Value	Effect Direction
rs60210535	14	46635410	*LINC00871*	G vs A	0.043 vs 0.047	.49	0.87 (0.59-1.28)	0.031 vs 0.050	9.0 × 10^-5^	0.58 (0.44-0.76)	.69	0.95 (0.76-1.20)	4.6 × 10^-5^	0.70 (0.59-0.83)	NA	NA	NA	NA
rs2622624	4	89069406	*ABCG2*	T vs C	0.385 vs 0.339	.08	1.16 (0.98-1.36)	0.372 vs 0.320	6.8 × 10^-5^	1.24 (1.11-1.37)	.24	1.11 (0.93-1.33)	.28	0.93 (0.81-1.06)	.59	+^c^	.003	+^c^

Abbreviations: CLHLS, Chinese Longitudinal Healthy Longevity Study; IDEAL, European Union Longevity Genetics Consortium; MAF, minor allele frequency; NA, not applicable; NECS, New England Centenarians Study; OR, odds ratio; SNP, single-nucleotide polymorphism.

^a^ The NECS had 801 centenarians (median age, 104 years) and 914 controls (mean age, 75 years).

^b^ The IDEAL had 7265 cases aged 85 years or older and 16 121 controls younger than 65 years from 14 studies in the Netherlands, Denmark, Iceland, Germany, Italy, United Kingdom, and Sweden. For this study, effect directions are available, but not ORs and 95% confidence intervals.

^c^ + indicates an allele that is more frequent in individuals aged 85 years or older compared with individuals younger than 65 years.

### Sex-Specific Pathway Analysis

Sex-specific differences were found in the biochemical pathways that influence human longevity. There are 11 pathways significantly associated with longevity in men (*P* < .005 and FDR < 0.05) (eTable 2 in the Supplement). These pathways are enriched mainly for immune and inflammatory responses, including immunity (TLR3) pathway, inflammatory cytokines and Toll-like receptor (TLR) signaling pathways, and the proinflammatory cytokine interleukin 6 (IL-6) pathway. In women, 34 pathways were enriched significantly (*P* < .005 and FDR < 0.05) and clustered to metabolic pathways (eTable 3 in the Supplement). The tryptophan metabolic pathway and the PPARγ coactivator-1α (PGC-1α) pathway were among the top pathways in this set.

### PRS Analyses to Assess Joint Effects of Groups of Sex-Specific Loci on Longevity

The PRS analyses using the north (or south) data set as the discovery sample and the south (or north) data set as the target sample showed that sex-specific joint associations with longevity of the 11 male-specific and 11 female-specific loci were replicated across north and south samples. More specifically, either using the north sample as the discovery and the south sample as the target, or vice versa, the 11 male-specific and 11 female-specific loci were jointly and significantly associated with longevity in one sex (*P* = 7.2 × 10^−22^ to 4.0 × 10^−12^) but not jointly associated with longevity in the other sex (*P* = .15-.76); the PRS-sex interaction effects were significant (*P* = 5.6 × 10^−20^ to 6.5 × 10^−8^) (Table 4).

**Table 4.** Polygenic Risk Score Analyses on the Joint Effects of Sex-Specific Loci’s Association With Longevity

Analysis	Main Effect of PRS in Men		Main Effect of PRS in Women		OR (95% CI) of PRS-Sex Interaction		*P *Value of PRS-Sex Interaction	Pseudo *R*^2^
	OR (95% CI)	*P * Value	OR (95% CI)	*P * Value	Men	Women		
A. Analyses using north data set as discovery sample and south data set as target sample^a^								
A1. 11 Male and 11 female loci with *P* < 10^-5^^b^								
11 Loci with *P* < 10^-5^ in men but *P* > .05 in women	2.136 (1.73-2.64)	4.0 × 10^-12^	1.040 (0.93-1.16)	.48	2.054 (1.62-2.61)	0.487 (0.38-0.62)	4.1 × 10^-9^	0.025
11 Loci with *P* < 10^-5^ in women but *P* > .05 in men	0.886 (0.75-1.05)	.15	1.782 (1.58-2.01)	4.1 × 10^-21^	0.497 (0.41-0.61)	2.011 (1.64-2.46)	2.2 × 10^-11^	0.040
A2. 35 Male and 25 female loci with 10^-5^ ≤ *P* < 10^-4^^c^								
35 Male-specific loci with 10^-5^ ≤ *P* < 10^-4^ in men but *P* > .25 in women	3.618 (2.86-4.58)	1.8 × 10^-26^	1.005 (0.90-1.12)	.92	3.599 (2.77-4.68)	0.278 (0.21-0.36)	8.5 × 10^-22^	0.066
25 Female-specific loci with 10^-5^ ≤ *P* < 10^-4^ in women but *P* > .35 in men	0.920 (0.78-1.09)	.33	2.229 (1.96-2.54)	2.5 × 10^-34^	0.413 (0.33-0.51)	2.423 (1.96-2.99)	2.2 × 10^-16^	0.069
B. Analyses using south data set as discovery sample and north data set as target sample								
B1. 11 Male and 11 female loci with *P* < 10^-5^								
11 Loci with *P* < 10^-5^ in men but *P* > .05 in women	2.473 (2.06-2.97)	7.2 × 10^-22^	0.935 (0.85-1.03)	.17	2.644 (2.15-3.25)	0.378 (0.31-0.46)	5.6 × 10^-20^	0.044
11 Loci with *P* < 10^-5^ in women but *P* > .05 in men	0.976 (0.84-1.14)	.76	1.626 (1.47-1.80)	1.3 × 10^-20^	0.601 (0.50-0.72)	1.665 (1.38-2.00)	6.5 × 10^-8^	0.037
B2. 35 Male and 25 female loci with 10^-5^ ≤ *P* < 10^-4^								
35 Male-specific loci with 10^-5^ ≤ *P* < 10^-4^ in men but *P* > .25 in women	3.509 (2.87-4.29)	4.2 × 10^-34^	0.956 (0.87-1.05)	.36	3.671 (2.93-4.59)	0.272 (0.22-0.34)	7.2 × 10^-30^	0.072
25 Female-specific loci with 10^-5^ ≤ *P* < 10^-4^ in women but *P* > .35 in men	0.872 (0.75-1.01)	.07	2.014 (1.80-2.25)	5.4 × 10^-35^	0.433 (0.36-0.52)	2.31 (1.92-2.78)	6.3 × 10^-19^	0.062
C. Analyses using north and south combined data set								
C1. 11 Male and 11 female loci with *P* < 10^-5^								
11 Loci with *P* < 10^-5^ in men but *P* > .05 in women	2.579 (2.24-2.97)	1.3 × 10^-39^	1.061 (0.99-1.14)	.11	2.431 (2.08-2.84)	0.411 (0.35-0.48)	1.0 × 10^-27^	0.043
11 Loci with *P* < 10^-5^ in women but *P* > .05 in men	0.978 (0.88-1.09)	.70	1.741 (1.61-1.88)	2.8 × 10^-42^	0.562 (0.49-0.64)	1.779 (1.55-2.04)	1.2 × 10^-16^	0.040
C2. 35 Male and 25 female loci with 10^-5^ ≤ *P* < 10^-4^								
35 Male-specific loci with 10^-5^ ≤ *P* < 10^-4^ in men but *P* > .25 in women	3.996 (3.40-4.69)	1.5 × 10^-64^	1.048 (0.97-1.13)	.21	3.812 (3.20-4.55)	0.262 (0.22-0.31)	4.8 × 10^-50^	0.079
25 Female-specific loci with 10^-5^ ≤ *P* < 10^-4^ in women but *P* > .35 in men	0.934 (0.84-1.04)	.22	2.141 (1.97-2.33)	2.8 × 10^-70^	0.436 (0.38-0.50)	2.293 (2.00-2.63)	4.3 × 10^-32^	0.066

Abbreviations: OR, odds ratio; PRS, polygenic risk score.

^a^ In analyses presented in sections A and B, we used the ORs of the sex-specific loci estimated based on the discovery sample of north (or south) data set as weights to construct the PRS scores in the target sample of south (or north) data set, following the standard procedure applied in the literature.^20^

^b^ The detailed information of the 11 male-specific and 11 female-specific longevity loci with *P* < 10^−5^ are presented in Tables 1 and 2.

^c^ The detailed information of the 35 male-specific and 25 female-specific loci with 10^−5 ^≤ *P* < 10^−4^ are presented in eTables 4 and 5 in the Supplement.

As discussed in eAppendix section S5 in the Supplement, based on the additional 47 male-specific and 34 female-specific candidate loci outlined earlier, we applied the stepwise approach that has been used widely in the PRS literature^18,20^ and we used the PRSice method and software^18^ to select an ideal *P* value cutoff (*P_T_*) in the other sex to provide the best-fitting PRS; we further identified 35 north-south individually replicated male-specific loci with *P* < 10^−4^ in men but *P* > .25 in women and 25 female-specific loci with *P* < 10^−4^ in women but *P* > .35 in men (eTables 4 and 5 in the Supplement). The results indicate that the sex-specific joint associations with longevity of these 35 male-specific and 25 female-specific loci were replicated across north and south samples; namely, they were jointly and significantly associated with longevity in one sex (*P* = 5.4 × 10^−35^ to 1.8 × 10^−26^) but not jointly associated with longevity in the other sex (*P* = .07-.93), and the PRS-sex interaction effects were significant (*P* = 2.2 × 10^−16^ to 7.2 × 10^−30^), either using the north sample as the discovery and the south as the target, or vice versa (Table 4).

Analyses using the north-south combined data set showed that the 11 male-specific and 11 female-specific loci (*P* < 10^−5^) and 35 male-specific and 25 female-specific loci (10^−5^ ≤ *P* < 10^−4^) were jointly and significantly associated with longevity in one sex (*P* = 2.9 × 10^−70^ to 1.3 × 10^−39^) but not jointly significant in the other sex (*P* = .11 to .70); PRS-sex interaction effects were significant (*P* = 4.8 × 10^−50^ to 1.2 × 10^−16^) (Table 4).

## Discussion

Of the 11 male-specific loci associated with longevity, rs1950902 in the *MTHFD1* gene is a nonsynonymous SNP that causes a C-to-T transition at nucleotide 401 resulting in an arginine-to-lysine substitution at amino acid 134 (C401T;R134K). *MTHFD1* was found to be associated with a protective role for colon and liver cancer risks prevalent in men^22^ and is consistent with the present study that *MTHFD1* is significantly and positively associated with longevity in men (*P* = 1.09 × 10^−7^) but not significant in women (*P* = .95) (Table 1).

Among the 11 male-specific loci associated with longevity, the SNP rs1027238 at *FAM19A1* was identified as a novel SNP that is significantly associated with longevity in women (*P* = 2.8 × 10^−6^) but not in men (*P* = .37). The SNP rs2161877 near *TBX3* was significantly associated with longevity in women (*P* = 2.9 × 10^−6^) but not in men (*P* = .72), which is consistent with previous findings that *TBX3* plays an important role in mammary gland development and breast cancer with a close relationship to estrogen.^23^

Clinical data demonstrate that men and women differ regarding their innate, humoral, and cell-mediated responses to bacterial and viral challenge.^24^ For example, men develop lower antibody responses and show significantly lower vaccine efficacy than women. Moreover, it is well known that longevity is associated with sex-specific differences in the immune system, and that there is a progressive decline in immunity and dysregulated inflammatory response in men.^25,26^ Consistent with these trends, and with previous genetics findings,^27,28^ we found that the proinflammatory cytokine IL-6 pathway was significantly associated with longevity in men. Furthermore, we found that the TLR3 signaling pathway was the most significant pathway associated with male longevity. Others have also reported that the TLR3 signaling pathway is dysregulated in elderly humans.^29^ TLR3 signaling evokes IL-6 production,^30^ and it initiates innate immunity and facilitates adaptive immunity by promoting maturation of dendritic cells.^30,31^ It is reasonable to hypothesize that dysregulation of the IL-6 and TLR3 signaling pathways renders men more susceptible than women to bacterial and viral infections; conversely, in long-lived men, altered IL-6 and TLR3 signaling pathways may provide greater protection against these challenges.^32^

Our findings regarding the female-specific tryptophan metabolic pathway reflect the documented significantly lower tryptophan levels in blood serum in female centenarians compared with the younger female controls (*P* < .001), but that the differences were not significant in male centenarians compared with younger male controls.^33^ Tryptophan metabolism contributes to a number of key processes, ranging from regulating innate and adaptive immunity^34^ to supporting intermediary metabolism via the provision of nicotinamide adenine dinucleotide (NAD^+^) and nicotinamide adenine dinucleotide phosphate to the biosynthesis of serotonin and related signaling molecules. PGC-1α is the master regulator of mitochondrial biogenesis and function because it promotes the expression of many of the more than 1000 nuclear-encoded mitochondrial genes and also participates in the regulation of innate immunity.^35^ One product of tryptophan metabolism, NAD^+^, is a cofactor for sirtuins, which have been implicated in inflammation, stress resistance, and aging. Coincidentally, sirtuin 1 deacetylates PGC-1α and enhances PGC-1α activity.^36^ Aging is associated with progressive mitochondrial dysfunction, and while the ultimate cause for this dysfunction is unknown, insufficient NAD^+^ availability and sirtuin 1 enzymatic activity may be contributing factors.^36,37^

In considering the female and male longevity-associated pathways together, the potential involvement of the innate immune system in men and of the tryptophan and PGC-1 pathways in the regulation of immune-related pathways in women suggests that women and men have optimized different approaches for solving the same biological riddle.

The estimates using QUANTO software version 1.1 (USC Biostats) indicate that both our male-specific GWAS and female-specific GWAS have acceptably good power (eTables 6a-6b in the Supplement). The estimates using the AVENGEME software^38^ indicate that power for both of our male-specific and female-specific PRS analyses is excellent: 0.997 to 0.999 for men and 1.00 for women (eTable 7 in the Supplement). As discussed in the Methods section, the sex-specific GWAS (stage 1) provides candidate sex-specific loci, and our conclusions of reconfirmed sex-specific longevity loci are mainly based on the PRS analyses (stage 2).

One may question whether the findings that loci that are significantly associated with longevity in women but not significant in men (Table 2 and Table 4; eTable 5 in the Supplement) are due to the substantially smaller sample size of male centenarians compared with female centenarians, which is common to all studies on longevity involving centenarians. We do not think this is the case because male centenarians are much more stringently mortality selected than their female counterparts, given that there were 2.3 male centenarians per 1 million men compared with 7.8 female centenarians per 1 million women in China in the 1990s,^39^ and the death rates in men were significantly higher than those of women at younger and older ages. Consequently, the *P* values of loci-sex interaction effects for male-specific loci (Table 1; eTable 4 in the Supplement) are all substantially smaller (ie, more significant) than the *P* values of loci-sex interaction effects for female-specific loci (Table 2; eTable 5 in the Supplement). These phenomena reflect a function of the greater mortality selection of survival to ages 100 years and older for the male centenarians than the female centenarians. Clearly, the male centenarians’ more stringent mortality selection may partially offset the shortage of power due to their much smaller sample size compared with female centenarians.

### Limitations

While our findings are innovative, the present study has some important limitations warranting further investigation. Unanswered questions include whether the genetic association with longevity is stronger in women or men and what the sex differences are in the genetic variants that are positively or negatively associated with longevity. More replications, meta-analyses, functional validations, and investigations of the effects of interactions between sex-specific genetic variants and environmental factors on health outcomes remain to be explored. Such further investigations may substantially contribute to more effective and targeted individualized health care for male and female elderly populations.

## Conclusions

The findings of the present study clearly indicate sex differences in genetic associations with longevity. Sex-specific associations with longevity of 4 exclusive groups of 11 male-specific and 11 female-specific loci (*P* < 10^−5^) and 35 male-specific and 25 female-specific loci (*P* < 10^−4^) are individually and jointly replicated across north and south discovery and target samples. Analyses using the north-south combined data set showed that these 4 groups of sex-specific loci are jointly and significantly associated with longevity in one sex (*P* = 2.9 × 10^−70^ to 1.3 × 10^−39^), but not jointly significant in the other sex (*P* = .11-.70), while interaction effects between PRS and sex are significant (*P* = 4.8 × 10^−50^ to 1.2 × 10^−16^). Although we recognize the large differences across ethnicities of different continents, it is noteworthy that 2 sex-specific loci were replicated between Chinese and US or European populations. We discovered that 11 male-specific pathways (inflammation and immunity genes) and 34 female-specific pathways (tryptophan metabolism and PGC-1α induced) are significantly associated with longevity.

As shown in Table 1 and Table 2 and eTables 4 and 5 in the Supplement, if one estimated regressions using the male-female combined data set adjusted for sex as a covariate without a loci-sex interaction term as used in all previously published GWAS on longevity,^2,3,4,5^ the *P* values of all of the north-south replicated sex-specific longevity loci listed in Table 1 and Table 2 and eTables 4 and 5 in the Supplement would increase substantially, and they would all become nonsignificant with the given suggestive significance level of *P* < 10^−5^ or* P* < 10^−4^, and 2 male-specific longevity loci (*P* < 10^−5^) in Table 1 and 11 male-specific longevity loci (*P* < 10^−4^) in eTable 4 in the Supplement would even have a *P* > .05. This is because the associations of the sex-specific loci with longevity are substantially offset by the nonsignificance in the other sex if the male-female combined data set were used while adjusted for sex as a covariate. As reviewed in the Introduction section, all previously published GWAS on longevity identified sex-independent genetic variants (such as *APOE, 5q33.3, IL6, FOXO1A, *and *FOXO3A*)^2,3,4,5,40^ but missed sex-specific loci and pathways associated with longevity. This is consistent with the conclusion that “genetic studies that ignore sex-specific effects in their design and interpretation could fail to identify a significant proportion of the genes that contribute to risk for complex diseases.”^41^ The present study contributes to filling this gap and identifies significant sex differences in genetic association with longevity.

Numerous studies have demonstrated sex differences in genetic variants’ reactions to the same nutritional intervention or drug treatment, steering away from the traditional view of one-size-fits-all health care and medicine.^42,43,44,45^ The present study provides a scientific basis for further investigations on sex-specific genetic variants associated with longevity and health to contribute to individualized health care. For example, the sex-specific loci and pathways significantly associated with longevity identified in the present study may serve as potential candidates of the sex-specific genomic biomarkers for in-depth research to be used in effective individualized health promotions and interventions.

## Notes

Supplement. **eAppendix. **Supplementary Material **eReferences** **eTable 1.** Sample Size and Ages of the Sex-Specific CLHLS GWAS Datasets **eTable 2.** The 11 Male-Specific Pathways Significantly Enriched and Associated With Longevity (*P*<0.005 and FDR<0.05) and the Significant Genes in These Pathways **eTable 3.** The 34 Female-Specific Pathways Significantly Enriched and Associated With Longevity (*P*<0.005 and FDR<0.05) and the Significant Genes in These Pathways **eTable 4.** The 35 Male-Specific Loci Associated With Longevity and Replicated in North and South Datasets, With a 10^-5^≤*P*<10^-4^ in Males but Not Significant in Females **eTable 5.** The 25 Female-Specific Loci Associated With Longevity and Replicated in North and South Datasets, With a 10^-5^≤*P*<10^-4^ in Females but Not Significant in Males **eTable 6.** Power Estimates for Male- and Female-Specific GWAS **eTable 7.** Parameters Used and Outcome of Power Estimates for the Sex-Specific PRS Analyses on Longevity Based on CLHLS GWAS Data, Using the AVENGEME Software **eFigure 1.** Manhattan Plots Showing Association of Longevity: Male **eFigure 2.** Manhattan Plots Showing Association of Longevity: Female **eFigure 3.** Quantile-Quantile Plots: Male **eFigure 4.** Quantile-Quantile Plots: Female
