# Supplementary material for: Sex Differences in Genetic Associations With Longevity
Source: JAMA Netw Open. 2018 Aug 24;1(4):e181670. doi: 10.1001/jamanetworkopen.2018.1670 (PMC6173523; doi:10.1001/jamanetworkopen.2018.1670)
Supplement: Supplement. — eAppendix. Supplementary Material eReferences eTable 1. Sample Size and Ages of the Sex-Specific CLHLS GWAS Datasets eTable 2. The 11 Male-Specific Pathways Significantly Enriched and Associated With Longevity (P<0.005 and FDR<0.05) and the Significant Genes in These Pathways eTable 3. The 34 Female-Specific Pathways Significantly Enriched and Associated With Longevity (P<0.005 and FDR<0.05) and the Significant Genes in These Pathways eTable 4. The 35 Male-Specific Loci Associated With Longevity and Replicated in North and South Datasets, With a 10-5≤P<10-4 in Males but Not Significant in Females eTable 5. The 25 Female-Specific Loci Associated With Longevity and Replicated in North and South Datasets, With a 10-5≤P<10-4 in Females but Not Significant in Males eTable 6. Power Estimates for Male- and Female-Specific GWAS eTable 7. Parameters Used and Outcome of Power Estimates for the Sex-Specific PRS Analyses on Longevity Based on CLHLS GWAS Data, Using the AVENGEME Software eFigure 1. Manhattan Plots Showing Association of Longevity: Male eFigure 2. Manhattan Plots Showing Association of Longevity: Female eFigure 3. Quantile-Quantile Plots: Male eFigure 4. Quantile-Quantile Plots: Female [file jamanetwopen-1-e181670-s001.pdf]

Supplementary Online Content

Zeng Y, Nie C, Min J, et al. Sex Differences in Genetic Associations With Longevity. *JAMA Netw Open*. 2018;1(4):e181670. doi:10.1001/jamanetworkopen.2018.1670

eAppendix. Supplementary Material
eReferences
eTable 1. Sample Size and Ages of the Sex-Specific CLHLS GWAS Datasets
eTable 2. The 11 Male-Specific Pathways Significantly Enriched and Associated With Longevity (P<0.005 and FDR<0.05) and the Significant Genes in These Pathways
eTable 3. The 34 Female-Specific Pathways Significantly Enriched and Associated With Longevity (P<0.005 and FDR<0.05) and the Significant Genes in These Pathways
eTable 4. The 35 Male-Specific Loci Associated With Longevity and Replicated in North and South Datasets, With a $10^{-5} \le P < 10^{-4}$ in Males but Not Significant in Females
eTable 5. The 25 Female-Specific Loci Associated With Longevity and Replicated in North and South Datasets, With a $10^{-5} \le P < 10^{-4}$ in Females but Not Significant in Males
eTable 6. Power Estimates for Male- and Female-Specific GWAS
eTable 7. Parameters Used and Outcome of Power Estimates for the Sex-Specific PRS Analyses on Longevity Based on CLHLS GWAS Data, Using the AVENGEME Software
eFigure 1. Manhattan Plots Showing Association of Longevity: Male
eFigure 2. Manhattan Plots Showing Association of Longevity: Female
eFigure 3. Quantile-Quantile Plots: Male
eFigure 4. Quantile-Quantile Plots: Female

This supplementary material has been provided by the authors to give readers additional information about their work.

© 2018 Zeng Y et al. *JAMA Network Open.*

eAppendix. Supplementary Material

S1. The case-control approach using centenarians as cases and middle-aged adults as controls

It is widely recognized that centenarian genomes may harbor genetic variants associated with health and longevity; thus, all of the GWAS on longevity published so far used centenarians (and/or nonagenarians aged 90+ or 85+) as cases and middle-aged adults as controls [1-9]. Case-control association analysis of longevity based on logistic regression is often used to estimate the association of the genetic variants with longevity by comparing the prevalence of the genetic variants between long-lived individuals and middle-aged controls [1-9]. This method, which uses long-lived individuals as cases and ethnically and geographically matched middle-aged persons as controls, is based on the fundamental demographic insight that the prevalence of a genetic variant in a population can change with age even though no individuals can change their fixed attribute of the genetic variant; therefore, much can be learned about the impact of the genetic variant on longevity. Ideally, we would use complete cohort data to compare the distributions of genetic variants of members of a cohort at two points of time in their life span: when they were young versus when some of them reach age 100+, while all of the other cohort members died before age 100. However, complete long-term follow-up GWAS or candidate gene genotype data are not currently available for members of the same birth cohort at young ages and ages 100+ for any population. Thus, all previously published case-control association studies of longevity used cross-sectional datasets to compare centenarians (and/or nonagenarians) as cases and middle-aged individuals as controls, both observed in the same time period [1-9]. Such cross-sectional case-control analyses are based on two assumptions: (a) The initial distribution of the genetic variants does not differ substantially between the long-lived and middle-aged cohorts, which is reasonable because the basic genetic structure would not change substantially in 40-50 years within the same ethnic population. (b) The basic genetic profile of the migrants does not differ substantially from non-migrants of the same ethnicity in the population studied. This assumption is also reasonable, especially for studies of Han Chinese because China has received remarkably few

© 2018 Zeng Y et al. *JAMA Network Open.*

immigrants in the past. With these two assumptions, one may intuitively understand that the proportion of genetic variants which are positively (or negatively) associated with longevity are significantly higher (or lower) among the centenarians compared to the middle-aged controls, because those who carry the longevity-favoring genetic variants have a better chance of surviving to age 100+, while those with less favorable genetic variants could not reach age 100. This logic relationship has been empirically shown [1-9] and proven mathematically [5].

Note that the odds ratios of a genotype's association with longevity are usually estimated with PLINK or other standard software using logistic regression based on the differences in proportions of individuals carrying the genotype among cases (centenarians/nonagenarians) and controls (middle-aged adults) which are determined by the effects of the genotype, gene-environment (GxE) interactions, and other factors related to the genotype. Thus the odds ratios cannot be interpreted as the size of pure effects of the genotype on longevity because the differences in proportions of carrying the genotype in the cases and controls also depend on other factors and GxE effects.

S2. Chinese Longitudinal Healthy Longevity Study (CLHLS)

The DNA samples and the data for the present study are from the CLHLS, which was conducted in 1998, 2000, 2002, 2005, 2008-2009, 2011-2012, 2014 and 2017-2018 in a randomly selected half of the counties and cities in 23 out of 31 provinces in China. The CLHLS covers approximately 85% of the total population of China. The CLHLS tried to interview all consenting centenarians in the sampled counties and cities. For each centenarian interviewee, compatible nearby un-related younger participants were interviewed, including one middle-aged adult aged 40-64, 1.5 young-old adult aged 65-79, one octogenarian aged 80-89 and one nonagenarian aged 90-99. "Nearby" is loosely defined – it could mean in the same village or the same street if available, or in the same town or in the same sampled county or city [10]. In the present study, we use centenarians as cases and middle-aged adults as controls; all of the DNA samples from middle-age controls were collected in the same county/city as the nearby centenarians [9].

© 2018 Zeng Y et al. *JAMA Network Open.*

Phenotype data were collected in the CLHLS using internationally standardized questionnaires adapted to the Chinese cultural and social context [10]. Extensive evaluations of the data quality of the CLHLS, including assessments of mortality rate, proxy use, non-response rate, sample attrition, reliability and validity of major health measures, and the rates of logically inconsistent answers, have shown that the data from the CLHLS surveys are of reasonably good quality [11-12]. The genetic samples and data from CLHLS were successfully used in prior published studies on candidate genes and gene-environment interactions associated with longevity [3-5, 13-14].

A wide variety of international and Chinese studies have confirmed that age reporting of the Han Chinese oldest-old aged 80+, including centenarians, is reasonably accurate [15-16]; this is due to the Han Chinese cultural tradition of memorizing one's date of birth to determine dates of important life events such as engagement, marriage, starting to build a residential house, etc. The accuracy of age reporting in the CLHLS data was reconfirmed by an investigation that compared standard demographic indices of age reporting, such as the single-age and sex distributions of the centenarians, the age progressive ratio among very old adults (e.g., aged 90 years or older), and centenarian density among the oldest-old, between the CLHLS and comparable data from Sweden, Japan, England and Wales, Australia, Canada, the U.S., and Chile; this study concluded that, although the quality of age reporting of Han Chinese centenarians was not as good as in Sweden, Japan, England and Wales, it was almost as good as in Australia and Canada, slightly better than the average of Whites and Black in the US, and much better than in Chile [17].

Note that the Han Chinese comprise about 93% of the total population in China, and 55 Chinese minority groups comprise the remaining 7% of the total population. The sample sizes of any minority group in the CLHLS data are too small for meaningful analysis, so we include Han Chinese samples only in the present study.

In the 1990s, there were approximately five centenarians per million people in China, compared with 50 per million in Western Europe in the same period [18]. Plausibly, Han Chinese centenarians may be more likely to have longevity-associated genes than their centenarian counterparts in the

© 2018 Zeng Y et al. *JAMA Network Open.*

Western world since they survived more brutal mortality regimes of the past when famine, wars, and starvation operated on birth cohorts of many millions. And unlike Western countries that received many international immigrants from other parts of the world resulting in relatively heterogeneous genetic compositions, even within the same ethnic group, China has received very few international immigrants. Consequently, Han Chinese are relatively more homogenous in genetic composition compared to their Western counterparts [19]. Thus, the present study of sex-specific genome-wide association study (GWAS) and polygenic risk score (PRS) analysis is expected to be instrumental for understanding sex differences in genetic associations with longevity.

S3. The genotype data (ref. to sections M2, M3 and M4 of our previously published article of CLHLS GWAS on longevity [9])

S3.1 Genotyping and quality control of the CLHLS GWAS datasets

The DNA samples of the centenarians and middle-aged controls used in the present study were genotyped by Beijing Genomics Institute (BGI) using the Illumina HumanOmniZhongHua-8 BeadChips, which was created by strategically selecting optimized tag SNP content from all three HapMap phases and the 1000 Genomes Project (1kGP). It allows profiling of 900,015 SNPs per sample, including 600k SNPs of common variants (MAF≥5%), 290k SNPs of rare variants (MAF<5%) and 10k SNPs existing only among Chinese and other Asian populations. In other words, 98.9% of the 900k SNPs of the Illumina HumanOmniZhongHua-8 BeadChip are internationally compatible, with 1.1% specific to Chinese and Asian populations; this provides coverage of about 81% of common variants at r^2 >0.8 with MAF ≥5%, and about 60% of rare variants at r^2 >0.8 with MAF<5%.

We randomly arranged cases and controls during sample batches, with negative controls (buffer water) and positive controls (YanHuang samples). For the sample filtering, 339 individuals for whom the genotypes were generated with a call rate less than 95% were excluded. The identity-by-state probabilities were also computed for all subjects to search for any possible duplicates and kinship-related individuals among the samples, using PLINK (1.06). The 134 individuals who had

© 2018 Zeng Y et al. *JAMA Network Open.*

identity-by-state probabilities with PI_HAT >0.25 were excluded based on the IBD analysis implemented in PLINK.

We performed principal components analysis (PCA) to evaluate genomic correlations in our dataset on the basis of pairwise identity by state (IBS) for all of the successfully genotyped samples using PLINK (1.06). We used the filter option (MAF >0.01 and genotyping rate >0.9) as discussed above; about 80% of the SNPs were used to calculate IBS. After excluding the 15 outliers, our PCA showed that the cases and controls in this study were of Han Chinese ancestry and were well matched, without evidence of gross population stratification. In general, cases and controls were not separated on the basis of the first two principal components, and the cases and controls are evenly distributed in clusters of PC1 vs. PC2, PC1 vs. PC3 and PC2 vs. PC3 (see Supplementary Fig. 1 of ref. 19). We computed the inflation factor (λ), a metric describing genome-wide inflation in association statistics [20], both before and after correcting for stratification using PLINK (1.06). Values of λ after correcting along 0, 1, 2, 3, or 4 eigenvectors are 1.109, 1.0241, 1.0235, 1.0226, or 1.0221, respectively, demonstrating that the correction is important and the top two eigenvectors correct nearly all of the stratification that can be corrected using the 4 eigenvectors. Consequently, in the subsequent GWAS regression models, we adjusted for the top two eigenvectors to minimize the effects of population stratification, following the approach adopted in the reference [21]. The genomic inflation factors (λ) in the South, North and combined datasets were 1.022, 1.010 and 1.022, respectively, indicating that the effects of population stratification on genetic analysis are well controlled [9]. The Manhattan plots and quantile-quantile plots for male/female and North/South datasets indicate that there is no inflation of the associations (eFigures 1-4).

Finally, after various stages of sample filtering, in total 4,477 of the CLHLS participants were included in the GWAS dataset (eTable 1), including 564 male and 1614 female centenarians (male mean age 101.5 with SD 3.58 and female mean age 103.1 with SD 3.41) and 773 male and 1526 female middle-aged controls (male mean age 48.7 with SD 6.68 and female mean age 48.2 with SD 7.79). The sample size of centenarians of the CLHLS GWAS dataset is the largest worldwide, 2.7

© 2018 Zeng Y et al. *JAMA Network Open.*

times as large as that of the next largest GWAS on longevity. We also conducted quality-control filtering of the GWAS data from the total 4,477 individuals. Following the standard adopted in the published studies of GWAS using the same 900k SNPs Illumina HumanOmniZhongHua-8 BeadChips [22] as the one used in the present study, SNPs with call rates of less than 90% were removed from our GWAS dataset; SNPs were also excluded if they had a MAF less than 1% or if there was significant deviation from Hardy-Weinberg equilibrium in the samples, defined as $P < 10^{-5}$. SNPs on the X and Y chromosomes and mitochondria were removed from the initial statistical analysis. Only 242 out of 743 (~30%) GWAS conducted from 2005 to 2011 considered chromosome X in their analyses [23]. Wise et al. [23] discussed several reasons for the exclusion of chromosome X, including a lower proportion of genes on chromosome X and a lower coverage of chromosome X on current genotyping platforms compared with autosomal coverage. They also described a number of technical hurdles that might add to the reluctance to include chromosome X in a GWAS, including complications in genotype calling, imputation, selection of test statistics, and the lack of readily available implementations. Accordingly, we excluded the chromosome X. After quality filtering and cleaning, 818,084 genotyped autosomal SNPs remained for association analysis in our final GWAS dataset.

To further increase genome coverage, we performed imputation analysis to infer the genotypes of all autosomal SNPs (MAF≥0.01) using IMPUTE software version 2 [24] and the 1000 Genomes Project integrated phase 1 release as reference panel. SNPs with a quality score (Rsq) of <0.9 were discarded before analysis. After standard GWAS quality-control filtering for subjects and SNPs as described above, we obtained data for 5,595,657 autosomal SNPs (818,084 genotyped SNPs and 4,777,573 imputed SNPs) in 2,178 centenarian cases and 2,299 middle-aged controls for the GWAS analyses.

S3.2 Stratifying South and North regions of China for discovery-evaluation analysis

China is considered to have geographically and traditionally distinct South and North regions that have existed for a few thousand years, although there is no legal or official administrative meaning to this

© 2018 Zeng Y et al. *JAMA Network Open.*

division. As reviewed in Xu et al. [19], previous analyses of anthropological, linguistic, and genetic data (including classic markers, microsatellite DNA markers, mtDNA, and Y chromosome SNP markers) have consistently suggested the presence of a significant boundary between the South and North populations in China. Among the 23 provinces where the CLHLS was conducted, Liaoning, Jilin, Heilongjiang, Hebei, Beijing, Tianjin, Shanxi, Shandong, Shaanxi, Jiangsu, Anhui and Henan belong to the geographically and traditionally defined North region; Shanghai, Zhejiang, Fujian, Jiangxi, Hubei, Hunan, Guangdong, Sichuan, Guangxi, Chongqing, and Hainan belong to the geographically and traditionally defined South region.

Our principle components analysis also genetically confirmed the geographic and traditional cultural division between South and North regions in the Han Chinese GWAS dataset (Supplementary Fig. 2 of ref. 19). Thus, we divided our GWAS datasets into four independent sub-datasets of male/ female and North/South region samples for discovery-evaluation analysis. The North region dataset consists of 286/829 male/female centenarians and 508/904 male/female middle-aged controls and the South region dataset consists of 278/785 male/female centenarians and 265/622 male/female middle-aged controls (eTable 1). Given the nature of our population analysis and due to the fact that all of the samples analyzed in this study belong to the same ethnic group of Han Chinese living in one country with the same culture, we also performed a combined analysis comparing all male/female centenarians with all male/female controls, respectively. The genomic inflation factors (λ) in the South and North datasets and the combined dataset were 1.022, 1.010 and 1.022, respectively, suggesting that the association test statistics conformed to the underlying null distribution and would not require further adjustment for genomic control.

S4. A bi-directional discovery-evaluation approach

In the traditional uni-directional discovery-evaluation approach, the entire sample is divided into two datasets, with one dataset used for discovery and the other dataset for replication or evaluation. The top Single nucleotide polymorphisms (SNPs) found in the discovery stage using a pre-determined *P*

© 2018 Zeng Y et al. *JAMA Network Open.*

value threshold are analyzed in the evaluation dataset. The SNPs with a *P* value lower than the threshold (e.g., $P < 10^{-4}$) in the discovery stage and nominal significance (e.g., $P < 0.05$) in the evaluation stage are identified as significant and replicated SNPs; the significant and replicated SNPs are then examined through association analysis using the discovery-evaluation combined dataset. This traditional uni-directional discovery-evaluation approach is especially useful when the expensive GWAS serves as the first stage of discovery and the much less expensive second stage of evaluation genotypes only the top SNPs with a *P* value lower than the threshold found in the discovery stage. However, when analyzing two available independent GWAS datasets, such as our present study, the uni-directional discovery-evaluation approach of fixing one GWAS dataset as discovery and another GWAS dataset as evaluation would have a much higher false-negative rate, missing a substantial number of significant and replicated SNPs which have a p-value higher than the threshold and lower than the nominal significance level in the fixed discovery GWAS dataset but reach the threshold significance level in the fixed evaluation GWAS dataset.

To avoid the high false-negative rate and to fully utilize all information in the sex-specific independent North and South GWAS datasets available to us, we applied a novel bi-directional discovery-evaluation approach [25] in our sex-specific GWAS and sex-specific PRS to identify the sex-specific loci significantly associated with longevity. Following this approach, we first analyzed the sex-specific North region GWAS dataset as discovery sample and the sex-specific South region GWAS dataset as evaluation sample (or target sample in sex-specific PRS analyses); we then reversed the process and analyzed the sex-specific South region GWAS dataset as discovery sample and the sex-specific North region GWAS dataset as evaluation (or target) sample. The results presented in Tables 1 and 2 indicate that our bi-directional approach identified 11 male-specific loci and 11 female-specific loci (in total 22 sex-specific loci) significantly associated with longevity in one sex but not significant in other sex and replicated across North and South regions. The results also show that if we adopted the classic uni-directional approach which fixes North as discovery and South as evaluation without reversing the process, 10 (45.5%) of the 22 sex-specific loci would be missed because they have a

© 2018 Zeng Y et al. *JAMA Network Open.*

$10^{-3} \leq P < 0.05$ in the North GWAS dataset and $P < 10^{-3}$ in the South GWAS dataset (the 10 loci which would be missed by the classic uni-directional approach include the last 3 loci in Table 1 and last 7 loci in Table 2). Clearly, our bi-directional approach substantially reduces the false-negative rate. At the same time, the overall false positive rate may increase as we identified more candidate sex-specific loci. However, this drawback is outweighed by the benefits, because the bi-directional discovery-evaluation approach substantially reduces the number of false negatives and creates a much more complete list of candidate sex-specific loci for further assessment in the sex-specific PRS analysis compared to the traditional uni-directional approach.

S5. The procedure of PRS analysis to identify truly sex-specific loci jointly associated with longevity using a more relaxed P_T threshold

As presented in Tables 1 and 2, we discovered 11/11 male/female specific longevity loci, which were replicated between North and South samples with a prior threshold of $P < 10^{-3}$ in the discovery step and had a $P < 10^{-5}$ in one sex but $P > 0.05$ in the other sex and $P < 0.05$ for the loci-sex interaction effects, using the North-South combined dataset. In addition to these findings, our sex-specific GWAS also found that 47/34 male/female loci, which were associated with longevity and replicated across North and South samples with a more relaxed prior threshold of $P < 0.01$ in the discovery step and had a $10^{-5} \leq P < 10^{-4}$ in one sex but $P > 0.05$ in the other sex and $P < 0.05$ for the loci-sex interaction effects, using the North-South combined dataset. We did not find any loci associated with longevity with a $P < 10^{-3}$ in both sexes but with effects in opposite directions.

The results presented in sections A1, B1 and C1 show that sex-specific joint associations with longevity of the 11/11 male/female loci were replicated across North and South samples; namely, they were jointly and highly significantly associated with longevity in one, but not jointly associated with longevity in the other sex; the PRS-sex interaction effects were highly significant, either using North sample as discovery and South as target, or vice versa. However, our PRS analysis indicated that the 47/34 male/female candidate loci are jointly and highly significantly associated with longevity with

© 2018 Zeng Y et al. *JAMA Network Open.*

$P<10^{-8}$ in one sex and also jointly and significantly associated with longevity in the other sex (P=0.007~0.001). Thus the groups of 47/34 male/female loci cannot be claimed as sex-specific longevity loci because they are also jointly and significantly associated with longevity in the other sex.

The joint and significant associations with longevity of these candidate loci in the other sex are because they also include some not-truly sex-specific loci, each of which is individually and slightly associated with longevity with a P larger than 0.05 and less than a P-threshold, but their joint effects are significant in the other sex. Thus, we need to filter and exclude those not-truly sex-specific loci from the 47/34 male/female loci in order to identify the groups of true sex-specific longevity loci. Following a trial and error approach for selecting an ideal P_T threshold to provide the best-fit PRS using the PRSice method and software [26], we selected the P threshold in the other sex to exclude the not-truly sex-specific loci. As shown in panels A2, B2 and C2 of Table 4, among the 47/34 male/female candidate loci, we identified 35/25 male/female specific loci which were also true sex-specific longevity loci, after filtering out the not-true sex-specific loci with the P_T threshold in the other sex identified by the PRSice. The North-South replicated 35 male-specific loci had a $10^{-5} \leq P < 10^{-4}$ in males but P >0.25 in females and P<0.05 for the loci-sex interaction effects; the North-South replicated 25 female-specific loci had a $10^{-5} \leq P < 10^{-4}$ in females but P >0.35 in males and P<0.05 for the loci-sex interaction effects.

We also tried but could not identify much larger sex-specific groups of loci with $P<10^{-3}$ or P<0.05, which are jointly and highly associated with longevity in one sex but are not jointly significant in the other sex and the sex-specific association replicated across North and South datasets. This is likely because inclusion of much large number of loci with $P<10^{-3}$ or P<0.05 could lead to overfitting -- adding complexity but increasing bias [27].

S6. Power estimates

Using the QUANTO (1.1) software, we evaluated the power of the sex-specific GWAS to detect genetic effects on longevity for samples of 564 male and 1,614 female centenarians and 773 male and

© 2018 Zeng Y et al. *JAMA Network Open.*

1,526 female middle-aged controls to achieve genome-wide significance (P=5×10^{-8}) and suggestive significance (P=5×10^{-7}, P=5×10^{-6}, P=5×10^{-5}, P=5×10^{-4}, P=5×10^{-3}) levels for different minor allele frequency (MAF), respectively. We used an additive genetic model and assumed that the prevalence of living to 100 years of age among male and female Han Chinese born in 1898-1908 is 2.33/10^6 and 7.83/10^6, respectively, based on estimates from previous study [18] and the ratio of male centenarians to female centenarians from the Chinese census [28]. The detailed power estimates results and parameters used are presented in eTables 6a-6b and indicate that our female-specific GWAS has a rather good power and the power of our male-specific GWAS is also acceptably good.

We conducted the power estimates for the sex-specific PRS analyses on longevity using the AVENGEME R program [29]. The detailed results and parameters used in the power estimates are presented in eTable 7 and indicate that the power for both male-specific and female-specific PRS analyses are excellent: 0.997~0.999 for males and 1.00 for females.

eReferences

1. Deelen J, Beekman M, Uh HW, et al. Genome-wide association meta-analysis of human longevity identifies a novel locus conferring survival beyond 90 years of age. *Hum Mol Genet*. 2014;23(16): 4420-4432.
2. Sebastiani P, Gurinovich A, Bae H, et al. Four Genome-Wide Association Studies Identify New Extreme Longevity Variants. *J Gerontol A Biol Sci Med Sci*. 2017: glx027.
3. Li Y, Wang WJ, Cao H, et al. Genetic association of FOXO1A and FOXO3A with longevity trait in Han Chinese populations. *Hum Mol Genet*. 2009;18(24):4897-4904.
4. Zhao L, Yang F, Xu K, et al. Common genetic variants of the β2-adrenergic receptor affect its translational efficiency and are associated with human longevity. *Aging cell*. 2012;11(6):1094-1101.
5. Zeng Y, Cheng L, Chen H, et al. Effects of FOXO genotypes on longevity: a biodemographic analysis. *J Gerontol A Biol Sci Med Sci*. 2010;65(12):1285-1299.
6. Newman AB, Walter S, Lunetta KL, et al. A meta-analysis of four genome-wide association studies of survival to age 90 years or older: the Cohorts for Heart and Aging Research in Genomic Epidemiology Consortium. *J Gerontol A Biol Sci Med Sci*. 2010;65(5):478-487.
7. Sebastiani P, Solovieff N, DeWan AT, et al. Genetic signatures of exceptional longevity in humans. *PloS one*. 2012;7(1):e29848.
8. Broer L, Buchman AS, Deelen J, et al. GWAS of longevity in CHARGE consortium confirms APOE and FOXO3 candidacy. *J Gerontol A Biol Sci Med Sci*. 2014;70(1):110-118.
9. Zeng Y, Nie C, Min J, et al. Novel loci and pathways significantly associated with longevity. *Sci Rep*. 2016;6:21243.
10. Zeng Y. Toward deeper research and better policy for healthy aging–using the unique data of

© 2018 Zeng Y et al. *JAMA Network Open.*

Chinese longitudinal healthy longevity survey. *China economic journal*. 2012;5(2-3):131-149.
11. Bongaarts J. Book review on "Zeng Yi, D.L.Poston, D. A. Vlosky,and Danan Gu (eds.), Healthy Longevity in China: Demographic, Socioeconomic, and Psychological Dimensions". *Population and Development Review.* 2009;35(2):452-453.
12. Goodkind D. Review on the book Healthy Longevity in China: Demographic, Socioeconomic, and Psychological Dimensions. *Population Studies.* 2009;63:1-7.
13. Zeng Y, Cheng L, Zhao L, et al. Interactions between Social/behavioral factors and ADRB2 genotypes may be associated with health at advanced ages in China. *BMC Geriatr*. 2013;13(1):91.
14. Zeng Y, Chen H, Ni T, et al. GxE Interactions between FOXO genotypes and tea drinking are significantly associated with cognitive disability at advanced ages in China. *J Gerontol A Biol Sci Med Sci*. 2014;70(4):426-433.
15. Coale AJ, Li S. The effect of age misreporting in China on the calculation of mortality rates at very high ages. *Demography.* 1991;28:293-301.
16. Wang Z, Zeng Y, Jeune B, Vaupel JW. Age validation of Han Chinese centenarians. *GENUS.* 1998;54:123–141.
17. Zeng Y, Gu D. Reliability of age reporting among the Chinese oldest-old in the CLHLS datasets. In: Zeng, Y. et al. (eds.). *Healthy longevity in China: Demographic, socioeconomic, and psychological dimensions.* pp.61-80. Dordrecht (The Netherlands: Springer Publisher). 2008.
18. Jeune, B. In search of the first centenarians. In: Jeune, B. & Vaupel, J. (eds.) Exceptional longevity: from prehistory to the present. Odense University Press, 1995. Pages 11–24.
19. Xu S, Yin X, Li S, et al. Genomic dissection of population substructure of Han Chinese and its implication in association studies. *Am J Hum Genet*. 2009;85(6):762-774.
20. Devlin B, Roeder K. Genomic control for association studies. *Biometrics.* 1999;55:997-100.
21. Price AL, Butler J, Patterson N, et al. Discerning the ancestry of European Americans in genetic association studies. *PLoS Genet*. 2008;4(1):e236.
22. Chen Y, Lin Y, Vithana EN, et al. Common variants near ABCA1 and in PMM2 are associated with primary open-angle glaucoma. *Nat Genet*. 2014;46(10):1115-1119.
23. Wise AL, Gyi L, Manolio TA. eXclusion: toward integrating the X chromosome in genome-wide association analyses. *Am J Hum Genet*. 2013;92(5):643-647.
24. Marchini J, Howie B, Myers S, McVean G, Donnelly P. A new multipoint method for genome-wide association studies by imputation of genotypes. *Nat. Genet*. 2007;39(7):906–913.
25. Jia P, Wang L, Fanous AH, et al. Network-assisted investigation of combined causal signals from genome-wide association studies in schizophrenia. *PLoS Comput Biol*. 2012;8(7): e1002587.
26. Euesden J, Lewis CM, O'Reilly PF. PRSice: polygenic risk score software. *Bioinformatics.* 2015;31(9):1466-1468.
27. Levine ME. Crimmins EM, Prescott CA, Phillips D, Arpawong TE, Lee J. A Polygenic Risk Score Associated with Measures of Depressive Symptoms Among Older Adults. *Biodemography Soc Biol*, 2014, 60:199–211.
28. National Bureau of Statistics (NBS) of the People's Republic of China. *Tabulations of the 2000 Census of China*. (China Statistics Press, Beijing).2002.
29. Dudbridge F. Power and predictive accuracy of polygenic risk scores. *PLoS Genet.* 2013;9(3): e1003348.

© 2018 Zeng Y et al. *JAMA Network Open.*

Supplement eTables

eTable 1. Sample size and ages of the sex-specific CLHLS GWAS datasets

	Males						Females					
	North region		South region		Total		North region		South region		Total	
	Centen -arians	Mid-age Controls	Centen -arians	Mid-age Controls	Centen -arians	Mid-age Controls	Centen -arians	Mid-age Controls	Centen -arians	Mid-age Controls	Centen -arians	Mid-age Controls
Sample size	286	508	278	265	564	773	829	904	785	622	1614	1526
Mean age	101.8	47.9	101.2	50.4	101.5	48.7	103.2	47.1	102.9	50	103.1	48.2
Standard deviation	3.24	6.54	3.70	6.64	3.48	6.68	3.44	7.38	3.37	8.05	3.41	7.79

eTable 2. The 11 *male-specific* pathways significantly enriched and associated with longevity (P<0.005 and FDR<0.05) and the significant genes in these pathways

Pathway/Gene set name	Descriptions of the male-specific pathways and the list of male-specific genes associated with longevity	*P*-value	FDR	Significant genes/ selected genes/All genes
CELLULAR BIOSYNTHETIC PROCESS	Genes annotated by the GO term GO:0044249. The chemical reactions and pathways resulting in the formation of substances, carried out by individual cells.<br>Significant genes:<br>IL6, FADS1, FADS2, TLR3, FARS2, GMDS, DMD, DAZL	< 0.001	0.0	8/23/321
GTPASE REGULATOR ACTIVITY	Genes annotated by the GO term GO:0030695. Modulates the rate of GTP hydrolysis by a GTPase.<br>Significant genes:<br>RGS6, RAPGEF6, RAPGEF4, ARHGEF3	< 0.001	0.0040	4/16/126
INFLAMMATORY RESPONSE	Genes annotated by the GO term GO:0006954. The immediate defensive reaction (by vertebrate tissue) to infection or injury caused by chemical or physical agents. The process is characterized by local vasodilation, extravasation of plasma into intercellular spaces and accumulation of white blood cells and macrophages.<br>Significant genes:<br>AOX1, BLNK, HDAC9, ADORA3, LY75	< 0.001	0.00525	5/14/130
TRANSLATION	Genes annotated by the GO term GO:0006412. The chemical reactions and pathways resulting in the formation of a protein. This is a ribosome-mediated process in which the information in messenger RNA (mRNA) is used to specify the sequence of amino acids in the protein.<br>Significant genes:<br>IL6, TLR3, FARS2, DAZL	< 0.001	0.005666667	4/14/180
BIOSYNTHETIC PROCESS	Genes annotated by the GO term GO:0009058. The energy-requiring part of metabolism in which simpler substances are transformed into more complex ones, as in growth and other biosynthetic processes.<br>Significant genes:<br>IL6, FADS1, FADS2, TLR3, FARS2, GMDS, ALG12, DMD, DAZL	< 0.001	0.0066666664	9/36/470
LIGASE ACTIVITY	Genes annotated by the GO term GO:0016874. Catalysis of the ligation of two substances with concomitant breaking of a diphosphate linkage, usually in a nucleoside triphosphate. Ligase is the systematic name for any enzyme of EC class 6.	< 0.001	0.0068000006	2/11/97

© 2018 Zeng Y et al. *JAMA Network Open.*

	Significant genes:<br>MTHFD1, FARS2			
MACROMOLECULE BIOSYNTHETIC PROCESS	Genes annotated by the GO term GO:0009059. The chemical reactions and pathways resulting in the formation of macromolecules, large molecules including proteins, nucleic acids and carbohydrates.<br>Significant genes:<br>IL6, TLR3, FARS2, GMDS, ALG12, DAZL,	< 0.001	0.009428571	6/27/321
ENZYME REGULATOR ACTIVITY	Genes annotated by the GO term GO:0030234. Modulates the activity of an enzyme.<br>Significant genes:<br>RGS6, RAPGEF6, RAPGEF4, SERPINB12, TWIST1, ARHGEF3	0.0020	0.03522222	6/33/324
RNA BINDING	Genes annotated by the GO term GO:0003723. Interacting selectively with an RNA molecule or a portion thereof.<br>Significant genes:<br>LRRFIP1, TLR3, FARS2, DAZL,	0.0010	0.038125	4/16/259
IMMUNE RESPONSE	Genes annotated by the GO term GO:0006955. Any immune system process that functions in the calibrated response of an organism to a potential internal or invasive threat.<br>Significant genes:<br>IL6, MAP3K7, BLNK, LY75	< 0.001	0.041100003	4/17/237
SMOOTH MUSCLE CONTRACTION	This pathway illustrates signaling networks implicated in uterine muscle contraction at labor and quiescence throughout gestation (pregnancy).<br>Significant genes:<br>IL6, RGS6, RGS7	0.0030	0.049909092	3/19/150

Note: The genes of MTHFD1 and LRRFIP1 which are part of the North-South replicated 11 male-specific longevity top loci (P<10^{-5}) (Table 1) contributed to the male-specific pathways of LIGASE ACTIVITY and RNA BINDING.

© 2018 Zeng Y et al. *JAMA Network Open.*

eTable 3. The 34 *female-specific* pathways significantly enriched and associated with longevity (P<0.005 and FDR<0.05) and the significant genes in these pathways

Pathway/Gene set name	Descriptions of the female-specific pathways and the list of female-specific genes associated with longevity	*P*-value	FDR	Significant genes/ Selected genes/All genes
TUBULIN BINDING	Genes annotated by the GO term GO:0015631. Interacting selectively with monomeric or multimeric forms of tubulin, including microtubules.<br>Significant genes:<br>NCALD, BIRC5, HTT, TUBGCP3	< 0.001	0.00925	4/14/47
TRYPTOPHAN METABOLISM	tryptophan => kynurenine => 2-aminomuconate<br>Significant genes:<br>CYP1A2, MAOB, ALDH3A2	< 0.001	0.009666666	3/11/56
RESPONSE TO NUTRIENT LEVELS	Genes annotated by the GO term GO:0031667. A change in state or activity of a cell or an organism (in terms of movement, secretion, enzyme production, gene expression, etc.) as a result of a stimulus reflecting the presence, absence, or concentration of nutrients.<br>Significant genes:<br>ENPP1, CCKAR, GHSR, ENSA	< 0.001	0.0108	4/11/29
VASCULATURE DEVELOPMENT	Genes annotated by the GO term GO:0001944. The process whose specific outcome is the progression of the vasculature over time, from its formation to the mature structure.<br>Significant genes:<br>FOXC2, RUNX1, AGGF1, CANX, NOTCH4, VEGFA	< 0.001	0.011333334	6/19/55
MICROTUBULE BINDING	Genes annotated by the GO term GO:0008017. Interacting selectively with microtubules, filaments composed of tubulin monomers.<br>Significant genes:<br>BIRC5, HTT	0.0020	0.0117500005	2/10/33
CATION BINDING	Genes annotated by the GO term GO:0043169. Interacting selectively with cations, charged atoms or groups of atoms with a net positive charge.<br>Significant genes:<br>KLF7, MSRB2, PCLO, BIRC5, ME1, APOBEC1, COMP, PITPNM3, CANX, ABL1, STIM1, NOTCH4	< 0.001	0.0124285715	12/61/213
PGC1APATHWAY	PCG-1a is expressed in skeletal muscle, heart muscle, and brown fat, and is a coactivator for receptors such as glucocorticoid receptor and thyroid hormone receptor.<br>Significant genes:<br>CAMK2D, PPARA, CAMK2A	0.0010	0.012727273	3/11/24
COAGULATION	Genes annotated by the GO term GO:0050817. The process by which a fluid solution, or part of it, changes into a solid or semisolid mass.<br>Significant genes:<br>ITGA2, HS3ST5, CD59, HNF4A, VWF	< 0.001	0.012846153	5/14/44
HSA04920 ADIPOCYTOKINE SIGNALING PATHWAY	Genes involved in adipocytokine signaling pathway<br>Significant genes:<br>PCK1, CAMKK1, TNFRSF1B, IRS4, PPARA	< 0.001	0.013	5/24/72
ANGIOGENESIS	Genes annotated by the GO term GO:0001525. Blood vessel formation when new vessels emerge from the proliferation of pre-existing blood vessels.<br>Significant genes:<br>RUNX1, AGGF1, CANX, NOTCH4, VEGFA	< 0.001	0.0133125	5/17/48
REGULATION OF BODY FLUID LEVELS	Genes annotated by the GO term GO:0050878. Any process that modulates the levels of body fluids.<br>Significant genes:	< 0.001	0.013333334	5/14/57

© 2018 Zeng Y et al. *JAMA Network Open.*

	ITGA2, CD59, HNF4A, VWF, FLI1			
HEMOSTASIS	Genes annotated by the GO term GO:0007599. The stopping of bleeding (loss of body fluid) or the arrest of the circulation to an organ or part.<br>Significant genes:<br>ITGA2, CD59, HNF4A, VWF, FLI1	< 0.001	0.013333334	5/14/48
ZINC ION BINDING	Genes annotated by the GO term GO:0008270. Interacting selectively with zinc (Zn) ions.<br>Significant genes:<br>KLF7, MSRB2, BIRC5, APOBEC1	< 0.001	0.0138	4/23/90
TRANSITION METAL ION BINDING	Genes annotated by the GO term GO:0046914. Interacting selectively with a transition metal ions; a transition metal is an element whose atom has an incomplete d-subshell of extranuclear electrons, or which gives rise to a cation or cations with an incomplete d-subshell. Transition metals often have more than one valency state. Biologically relevant transition metals include vanadium, manganese, iron, copper, cobalt, nickel, molybdenum and silver.<br>Significant genes:<br>KLF7, MSRB2, BIRC5, ME1, APOBEC1, ABL1	< 0.001	0.014	6/27/111
SMALL GTPASE REGULATOR ACTIVITY	Genes annotated by the GO term GO:0005083. Modulates the rate of GTP hydrolysis by a small monomeric GTPase.<br>Significant genes:<br>RAPGEF5, TIAM1, ARHGDIA, PREX1, ARHGEF1	< 0.001	0.016	5/17/67
MITOCHONDRIAL ENVELOPE	Genes annotated by the GO term GO:0005740. The double lipid bilayer enclosing the mitochondrion and separating its contents from the cell cytoplasm; includes the intermembrane space.<br>Significant genes:<br>NDUFS2, MAOB, MCL1	0.001	0.016529411	3/12/97
ORGAN MORPHOGENESIS	Genes annotated by the GO term GO:0009887. Morphogenesis of an organ. An organ is defined as a tissue or set of tissues that work together to perform a specific function or functions. Morphogenesis is the process by which anatomical structures are generated and organized. Organs are commonly observed as visibly distinct structures, but may also exist as loosely associated clusters of cells that work together to perform a specific function or functions.<br>Significant genes:<br>ITGA2, HTT, RUNX1, COMP, AGGF1, ENG, CANX, COL9A1, FLI1, NOTCH4, TBX3	< 0.001	0.017052632	11/50/145
POSITIVE REGULATION OF DEVELOPMENTAL PROCESS	Genes annotated by the GO term GO:0051094. Any process that activates or increases the rate or extent of development, the biological process whose specific outcome is the progression of an organism over time from an initial condition (e.g. a zygote, or a young adult) to a later condition (e.g. a multicellular animal or an aged adult).<br>Significant genes:<br>APOE, SAP30BP, HTT, IFI16, ROBO2, RUNX1, ADAMTSL4, AGGF1, SMAD3, ABL1, TNFSF10, MAP3K10, DAPK1	< 0.001	0.017222222	13/61/218
PROTEIN HETERODIMERIZATION ACTIVITY	Genes annotated by the GO term GO:0046982. Interacting selectively with a nonidentical protein to form a heterodimer.<br>Significant genes:<br>APOE, BIRC5, PRMT8, PDSS2, NOTCH4, RAB3GAP2, MCL1	< 0.001	0.017590908	7/27/77
PROTEIN DIMERIZATION ACTIVITY	Genes annotated by the GO term GO:0046983. The formation of a protein dimer, a macromolecular structure consists of two noncovalently associated identical or nonidentical subunits.	< 0.001	0.017695652	15/64/182

© 2018 Zeng Y et al. *JAMA Network Open.*

	Significant genes:<br>ABCG2, PVRL2, APOE, BIRC5, WWOX, SMAD3, VWF, PRMT8, PDSS2, NOTCH4, RAB3GAP2, MAP3K10, MCL1, ABAT, FOXP2			
HSA00340 HISTIDINE METABOLISM	Genes involved in histidine metabolism<br>Significant genes:<br>UROC1, PRMT8, MAOB, ALDH3A2	< 0.001	0.017904762	4/11/41
RESPONSE TO EXTRACELLULAR STIMULUS	Genes annotated by the GO term GO:0009991. A change in state or activity of a cell or an organism (in terms of movement, secretion, enzyme production, gene expression, etc.) as a result of an extracellular stimulus.<br>Significant genes:<br>ENPP1, CCKAR, GHSR, ENSA	< 0.001	0.018041667	4/12/33
GUANYL NUCLEOTIDE EXCHANGE FACTOR ACTIVITY	Genes annotated by the GO term GO:0005085. Stimulates the exchange of guanyl nucleotides by a GTPase. Under normal cellular physiological conditions, the concentration of GTP is higher than that of GDP, favoring the replacement of GDP by GTP in association with the GTPase.<br>Significant genes:<br>RAPGEF5, TIAM1, PREX1, ARHGEF1, FGD6	< 0.001	0.01805	5/17/47
RIBONUCLEASE ACTIVITY	Genes annotated by the GO term GO:0004540. Catalysis of the hydrolysis of phosphodiester bonds in chains of RNA.<br>Significant genes:<br>RNASE2, POP1, EXOSC2	0.0020	0.01988	3/10/25
HSA00380 TRYPTOPHAN METABOLISM	Genes involved in tryptophan metabolism<br>Significant genes:<br>CYP1A2, LNX1, AFMID, PRMT8, MAOB, ALDH3A2	< 0.001	0.022	6/20/60
ORGANELLE ENVELOPE	Genes annotated by the GO term GO:0031967. A double membrane structure enclosing an organelle, including two lipid bilayers and the region between them. In some cases, an organelle envelope may have more than two membranes.<br>Significant genes:<br>NDUFS2, GRINL1A, NUP133, NUP153, MAOB, TRIM27, MCL1	< 0.001	0.022555556	7/28/169
ENVELOPE	Genes annotated by the GO term GO:0031975. A multilayered structure surrounding all or part of a cell; encompasses one or more lipid bilayers, and may include a cell wall layer, also includes the space between layers.<br>Significant genes:<br>NDUFS2, GRINL1A, NUP133, NUP153, MAOB, TRIM27, MCL1	< 0.001	0.022555556	7/28/169
BLOOD COAGULATION	Genes annotated by the GO term GO:0007596. The sequential process by which the multiple coagulation factors of the blood interact, ultimately resulting in the formation of an insoluble fibrin clot; it may be divided into three stages: stage 1, the formation of intrinsic and extrinsic prothrombin converting principle; stage 2, the formation of thrombin; stage 3, the formation of stable fibrin polymers<br>Significant genes:<br>ITGA2, CD59, HNF4A, VWF	0.0010	0.031535715	4/13/43
ION BINDING	Genes annotated by the GO term GO:0043167. Interacting selectively with ions, charged atoms or groups of atoms.<br>Signiicant genes:<br>KLF7, MSRB2, PCLO, BIRC5, ME1, APOBEC1, COMP, PITPNM3, CANX, ABL1, STIM1, NOTCH4, TRIM27	< 0.001	0.0334	13/74/274
NEGATIVE REGULATION OF MULTICELLULAR	Genes annotated by the GO term GO:0051241. Any process that stops, prevents or reduces the frequency, rate or extent of an organismal process, the processes pertinent to the	0.0040	0.033724137	2/11/32

© 2018 Zeng Y et al. *JAMA Network Open.*

ORGANISMAL PROCESS	function of an organism above the cellular level; includes the integrated processes of tissues and organs.<br>Significant genes:<br>HS3ST5, GHSR			
REGULATION OF APOPTOSIS	Genes annotated by the GO term GO:0042981. Any process that modulates the occurrence or rate of cell death by apoptosis.<br>Significant genes:<br>APOE, BIRC5, SAP30BP, HTT, IFI16, IL2RB, ADAMTSL4, SMAD3, ARHGDIA, ABL1, CFL1, TNFSF10, TAX1BP1, DNAJB6, VEGFA, MA P3K10, DAPK1, MCL1	0.0010	0.03951613	18/88/342
ANATOMICAL STRUCTURE FORMATION	Genes annotated by the GO term GO:0048646. The process pertaining to the initial formation of an anatomical structure from unspecified parts. This process begins with the specific processes that contribute to the appearance of the discrete structure and ends when the structural rudiment is recognizable. An anatomical structure is any biological entity that occupies space and is distinguished from its surroundings. Anatomical structures can be macroscopic such as a carpel, or microscopic such as an acrosome.<br>Significant genes:<br>RUNX1, AGGF1, CANX, NOTCH4, VEGFA	0.0020	0.04063636	5/22/56
REGULATION OF PROGRAMMED CELL DEATH	Genes annotated by the GO term GO:0043067. Any process that modulates the frequency, rate or extent of programmed cell death, cell death resulting from activation of endogenous cellular processes.<br>Significant genes:<br>APOE, BIRC5, SAP30BP, HTT, IFI16, IL2RB, ADAMTSL4, SMAD3, ARHGDIA, ABL1, CFL1, TNFSF10, TAX1BP1, DNAJB6, VEGFA, MAP3K10, DAPK1, MCL1	0.0010	0.040705882	18/89/343
REGULATION OF DEVELOPMENTAL PROCESS	Genes annotated by the GO term GO:0050793. Any process that modulates the frequency, rate or extent of development, the biological process whose specific outcome is the progression of a multicellular organism over time from an initial condition (e.g. a zygote, or a young adult) to a later condition (e.g. a multicellular animal or an aged adult).<br>Significant genes:<br>APOE, BIRC5, SAP30BP, HTT, IFI16, IL2RB, ROBO2, RUNX1, ADAMTSL4, AGGF1, SMAD3, ARHGDIA, ABL1, CFL1, TNFSF10, TAF8, HDAC9, TAX1BP1, DNAJB6, NOTCH4, VEGFA, MAP3K10, DAPK1, MCL1, FGD6	< 0.001	0.04165625	25/132/442

Note: The NOTCH4 and TBX3 genes which are part of the North-South replicated 11 female-specific longevity top loci ($P<10^{-5}$) (Table 2) contributed to multiple female-specific pathways of VASCULATURE DEVELOPMENT, CATION BINDING, ANGIOGENESIS, ORGAN MORPHOGENESIS, PROTEIN HETERODIMERIZATION ACTIVITY, PROTEIN DIMERIZATION ACTIVITY, ION BINDING, ANATOMICAL STRUCTURE FORMATION, and REGULATION OF DEVELOPMENTAL PROCESS.

© 2018 Zeng Y et al. *JAMA Network Open.*

eTable 4. The 35 *male-specific loci* associated with longevity and replicated in North and South datasets, with a $10^{-5} \leqslant P < 10^{-4}$ in males but not significant in females

loci	Chr	Nearby gene	(A) *Males* North and South discovery-evaluation analysis								(B) *Females* (North-South combined)			(C) Regression with Loci-sex interaction term			(D) Regression adjusted for sex as a covariate without loci-sex interaction term, using males-females and North-South combined dataset	
			(A-1) *Males* (North)		(A-2) *Males* (South)		(A-3) *Males* (North-South Combined)							OR of loci-sex interaction (North-South combined)		*P* of loci-sex Interact. effects		
			P	OR	*P*	OR	MAF(case /control)	*P*	*FDR*	OR	MAF(case /control)	*P*	OR	loci x male	loci x female		*P*	OR
***P*<0.01 in North males and *P*<0.05 in South males, but *P>0.05* in females**																		
rs2526625	7	HDAC9	2.48E-04	1.722	4.44E-02	1.295	0.255/0.188	5.92E-05	1.15E-04	1.470	0.226/0.227	0.724	0.979	1.501	0.666	3.22E-04	0.057	1.101
rs3822311	5	RAPGEF6	5.49E-04	2.033	1.28E-02	1.531	0.130/0.076	2.76E-05	3.22E-04	1.724	0.098/0.096	0.875	1.013	1.701	0.588	6.09E-04	0.015	1.187
rs73146320	3	DAZL	6.21E-04	0.637	1.15E-02	0.737	0.264/0.330	5.76E-05	1.19E-04	0.700	0.316/0.302	0.340	1.054	0.664	1.505	8.99E-05	0.184	0.940
rs71547564	7	ESYT2	9.23E-04	1.556	5.88E-03	1.370	0.489/0.413	1.38E-05	4.82E-04	1.456	0.440/0.436	0.578	1.030	1.413	0.708	6.80E-04	0.006	1.133
rs2881564	17	ACCN1	1.01E-03	1.569	2.21E-02	1.307	0.323/0.242	7.52E-05	1.20E-04	1.418	0.288/0.282	0.976	0.998	1.420	0.704	8.15E-04	0.034	1.105
rs11949251	5	FTMT	1.06E-03	0.566	1.30E-02	0.660	0.117/0.167	3.63E-05	1.82E-04	0.608	0.150/0.151	0.643	0.968	0.628	1.591	8.76E-04	0.011	0.858
rs6826101	4	GPR125	1.20E-03	0.499	1.57E-02	0.632	0.069/0.116	3.97E-05	1.54E-04	0.557	0.096/0.095	0.928	1.008	0.552	1.810	3.89E-04	0.027	0.851
rs4851975	2	HDAC4	1.37E-03	1.548	3.30E-03	1.408	0.450/0.359	1.51E-05	2.64E-04	1.465	0.407/0.401	0.645	1.025	1.429	0.700	5.66E-04	0.005	1.136
rs1563914	8	LOC286114	1.39E-03	1.683	6.13E-03	1.435	0.229/0.167	3.70E-05	1.62E-04	1.519	0.199/0.204	0.893	0.992	1.532	0.653	3.47E-04	0.027	1.124
rs4441708	4	GDEP	2.01E-03	0.665	6.10E-03	0.718	0.260/0.331	4.74E-05	1.27E-04	0.697	0.304/0.291	0.297	1.060	0.657	1.522	6.44E-05	0.170	0.938
rs2681748	14	RGS6	2.06E-03	1.479	9.98E-03	1.328	0.506/0.413	2.77E-05	2.42E-04	1.414	0.445/0.439	0.762	1.017	1.391	0.719	8.45E-04	0.013	1.118
rs10015331	4	PCDH18	2.40E-03	0.688	3.92E-03	0.733	0.402/0.483	8.06E-05	1.13E-04	0.729	0.419/0.430	0.504	0.967	0.754	1.326	2.99E-03	0.004	0.885
rs6765952	3	CNTN6	2.43E-03	0.690	1.37E-02	0.757	0.382/0.457	9.17E-05	1.15E-04	0.724	0.434/0.426	0.604	1.028	0.704	1.420	3.61E-04	0.092	0.928
rs60188330	15	RORA	2.62E-03	1.590	4.12E-03	1.472	0.229/0.160	4.28E-05	1.50E-04	1.509	0.203/0.203	0.789	0.983	1.535	0.652	2.98E-04	0.039	1.115
rs10092258	8	SNTB1	3.18E-03	1.427	8.31E-03	1.332	0.471/0.395	9.99E-05	9.99E-05	1.367	0.441/0.429	0.352	1.049	1.303	0.767	5.46E-03	0.003	1.134
rs72794530	16	MIR4719	4.35E-03	1.685	4.96E-03	1.551	0.159/0.105	5.62E-05	1.23E-04	1.608	0.122/0.124	0.746	0.975	1.650	0.606	4.20E-04	0.051	1.135
rs17046973	2	DDX18	5.97E-03	0.700	5.61E-03	0.722	0.288/0.347	9.90E-05	1.02E-04	0.714	0.318/0.321	0.530	0.966	0.739	1.354	3.19E-03	0.008	0.884
rs11713260	3	FGF12	6.30E-03	0.710	5.67E-03	0.736	0.340/0.419	9.21E-05	1.11E-04	0.724	0.369/0.370	0.897	0.993	0.729	1.372	1.20E-03	0.018	0.901
rs726890	7	SH2B2	7.48E-03	0.708	1.53E-03	0.703	0.318/0.403	4.58E-05	1.34E-04	0.711	0.354/0.355	0.797	1.014	0.701	1.426	3.34E-04	0.039	0.913
rs1511386	3	ACPL2	8.86E-03	0.589	2.35E-03	0.552	0.074/0.122	4.36E-05	1.39E-04	0.564	0.098/0.106	0.315	0.919	0.613	1.631	2.81E-03	0.002	0.805
***P*<0.01 in South males and *P*<0.05 in North males, but *P>0.05* in females**																		
rs79618463	3	MIR548AC	1.10E-02	3.074	6.73E-03	2.813	0.036/0.012	7.57E-05	1.15E-04	3.089	0.020/0.019	0.737	1.064	2.904	0.344	1.64E-03	0.010	1.476
rs8045471	16	RMI2	1.26E-02	1.376	1.58E-03	1.413	0.399/0.323	5.52E-05	1.29E-04	1.396	0.335/0.344	0.360	0.953	1.465	0.682	1.02E-04	0.209	1.057
rs886945	12	CACNA2D4	1.44E-02	0.568	3.03E-03	0.576	0.069/0.117	8.93E-05	1.16E-04	0.568	0.103/0.097	0.443	1.068	0.532	1.879	1.72E-04	0.143	0.900
rs11200558	10	BTBD16	2.06E-02	0.735	8.90E-04	0.661	0.238/0.308	9.86E-05	1.05E-04	0.704	0.287/0.286	0.949	0.996	0.706	1.416	1.08E-03	0.034	0.905
rs73244783	8	FAM110B	2.16E-02	1.445	6.59E-04	1.640	0.192/0.128	5.10E-05	1.27E-04	1.545	0.152/0.159	0.253	0.922	1.675	0.597	6.17E-05	0.205	1.077
rs139013	22	SCUBE1	2.44E-02	1.325	1.23E-03	1.404	0.523/0.449	6.74E-05	1.18E-04	1.375	0.479/0.493	0.395	0.958	1.436	0.696	1.35E-04	0.128	1.067
rs10887732	10	PAPSS2	2.50E-02	1.364	8.22E-04	1.485	0.498/0.423	8.36E-05	1.13E-04	1.422	0.471/0.461	0.409	1.047	1.359	0.736	3.63E-03	0.004	1.145
rs11188709	10	DNTT	3.09E-02	1.646	1.25E-03	1.900	0.100/0.058	7.74E-05	1.13E-04	1.812	0.085/0.081	0.454	1.073	1.688	0.592	3.19E-03	0.004	1.258
rs11241767	5	ZNF608	3.21E-02	0.745	6.65E-04	0.663	0.246/0.322	9.61E-05	1.09E-04	0.704	0.284/0.292	0.688	0.977	0.720	1.388	2.15E-03	0.014	0.889
rs140690	18	MBD1	3.50E-02	0.656	1.62E-03	0.585	0.094/0.148	9.43E-05	1.10E-04	0.605	0.120/0.129	0.346	0.929	0.651	1.537	4.39E-03	0.003	0.825
rs80155229	3	DOCK3	3.65E-02	1.411	1.54E-04	1.800	0.199/0.131	3.20E-05	1.87E-04	1.599	0.167/0.156	0.555	1.043	1.533	0.652	1.34E-03	0.008	1.173

© 2018 Zeng Y et al. *JAMA Network Open.*

rs4658871	1	TRIM67	3.88E-02	1.298	7.59E-04	1.483	0.359/0.283	6.88E-05	1.15E-04	1.406	0.307/0.312	0.509	0.964	1.458	0.686	2.18E-04	0.117	1.075
rs10797073	1	SLAMF6	3.93E-02	1.313	6.00E-04	1.485	0.417/0.339	9.71E-05	1.06E-04	1.401	0.390/0.382	0.757	1.017	1.378	0.726	1.64E-03	0.017	1.115
rs272659	7	STK31	3.99E-02	0.774	2.65E-04	0.665	0.384/0.460	3.06E-05	2.14E-04	0.708	0.429/0.415	0.317	1.053	0.672	1.488	4.78E-05	0.147	0.939
rs9302081	13	CLDN10	4.37E-02	0.664	8.91E-04	0.534	0.076/0.128	6.14E-05	1.13E-04	0.577	0.100/0.105	0.458	0.939	0.614	1.628	2.54E-03	0.004	0.813

Notes: (1) In panel (C), the estimates of *P* of (loci x sex) interaction effects are based on logistic regressions including the loci, sex and (loci x sex) interaction term, using the North-South combined dataset; the estimates of *P* and OR of the main effects of the loci in males and females are not presented in panel (C) because they are almost exactly the same as the *P* and OR of the loci in panels (A-3) and (B), respectively, estimated based on the male and female datasets separately, which are statistically expected.

© 2018 Zeng Y et al. *JAMA Network Open.*

eTable 5. The 25 *female-specific loci* associated with longevity and replicated in North and South datasets, with a $10^{-5} \leqslant P < 10^{-4}$ in females but not significant in males

loci	Chr	Nearby gene	(D) *Females* North and South discovery-evaluation analysis								(B) *Males* (North-South combined)			(C) Regression with loci-sex interaction term			(D) Regression adjusted for sex as a covariate without loci-sex interaction term, using males-females and North-South combined dataset	
			(A-1) *Females* (North)		(A-2) *Females* (South)		(A-3) *Females* (North-South Combined)							OR of loci-sex interaction (North-South combined)		*P* of loci-sex Interact. effects		
			P	OR	*P*	OR	MAF(case /control)	*P*	*FDR*	OR	MAF(case /control)	*P*	OR	loci x male	loci x female		*P*	OR
***P*<0.01 in North females and *P*<0.05 in South females, but *P>0.05* in males**																		
rs1065652	10	MAP3K8	3.56E-04	0.745	3.17E-02	0.839	0.241/0.280	4.25E-05	1.33E-04	0.789	0.250/0.231	0.524	1.061	1.346	0.743	0.007	2.44E-03	0.862
rs137973757	2	C2orf44	3.98E-04	0.288	4.85E-02	0.457	0.007/0.019	8.96E-05	1.02E-04	0.358	0.020/0.015	0.567	1.197	3.344	0.299	0.003	2.31E-03	0.551
rs12411233	1	PRDM16	6.36E-04	1.707	6.35E-03	1.403	0.100/0.069	1.52E-05	3.81E-04	1.517	0.073/0.081	0.364	0.871	0.574	1.743	0.002	9.13E-04	1.303
rs3001223	1	CDC73	6.40E-04	0.732	2.45E-02	0.838	0.225/0.269	7.61E-05	1.19E-04	0.790	0.241/0.243	0.919	1.009	1.277	0.783	0.024	1.29E-03	0.852
rs10826172	10	UBE2D1	7.48E-04	0.758	1.48E-02	0.836	0.416/0.469	3.28E-05	2.05E-04	0.798	0.451/0.449	0.890	1.012	1.269	0.788	0.017	5.13E-04	0.854
rs145656202	4	FSTL5	1.02E-03	0.354	2.16E-02	0.472	0.009/0.022	8.02E-05	1.18E-04	0.408	0.015/0.014	0.932	0.971	2.382	0.420	0.034	7.16E-04	0.530
rs154740	21	CHODL	1.92E-03	0.547	1.11E-02	0.665	0.037/0.059	9.63E-05	1.00E-04	0.618	0.040/0.043	0.946	0.986	1.596	0.627	0.048	9.86E-04	0.707
rs58730464	7	LOC100134229	2.05E-03	0.786	1.03E-02	0.835	0.388/0.436	6.35E-05	1.32E-04	0.812	0.398/0.402	0.920	0.992	1.222	0.818	0.034	8.30E-04	0.865
rs10146989	14	NID2	2.24E-03	1.275	7.46E-03	1.212	0.429/0.380	5.50E-05	1.25E-04	1.239	0.414/0.409	0.834	1.017	0.821	1.218	0.039	6.18E-04	1.162
rs686237	20	B4GALT5	2.34E-03	1.277	5.59E-03	1.215	0.495/0.444	8.33E-05	1.16E-04	1.230	0.455/0.451	0.910	1.009	0.821	1.219	0.043	4.13E-04	1.169
rs7793753	7	MIR148A	2.50E-03	0.783	5.26E-03	0.819	0.315/0.363	4.14E-05	1.48E-04	0.803	0.346/0.328	0.434	1.066	1.328	0.753	0.004	2.81E-03	0.875
rs61893562	11	SESN3	4.04E-03	0.692	3.31E-03	0.728	0.103/0.136	4.99E-05	1.39E-04	0.716	0.120/0.122	0.984	0.998	1.393	0.718	0.029	6.53E-04	0.791
rs2701153	12	FAM19A2	4.06E-03	0.795	8.98E-03	0.832	0.430/0.482	8.60E-05	1.13E-04	0.813	0.461/0.459	0.914	0.991	1.219	0.820	0.042	8.08E-04	0.863
rs4134803	15	CYFIP1	5.37E-03	0.805	1.13E-03	0.795	0.423/0.476	1.93E-05	2.42E-04	0.800	0.433/0.423	0.676	1.035	1.293	0.773	0.008	1.25E-03	0.868
rs10777685	12	MIR3685	6.61E-03	1.271	4.35E-03	1.241	0.307/0.264	9.87E-05	9.87E-05	1.250	0.278/0.296	0.362	0.923	0.738	1.355	0.004	5.99E-03	1.140
rs1363846	5	PARP8	6.66E-03	1.398	4.44E-03	1.322	0.146/0.114	5.33E-05	1.33E-04	1.362	0.132/0.140	0.720	0.960	0.705	1.419	0.010	1.67E-03	1.217
rs76354584	14	FLRT2	7.71E-03	1.523	2.62E-03	1.450	0.091/0.066	7.50E-05	1.25E-04	1.467	0.073/0.079	0.805	0.964	0.657	1.522	0.018	1.13E-03	1.297
rs3732736	3	EIF4E3	8.67E-03	0.817	3.74E-03	0.815	0.397/0.446	8.81E-05	1.05E-04	0.816	0.453/0.445	0.639	1.038	1.271	0.787	0.011	1.43E-03	0.872
rs12153373	5	LOC285627	9.12E-03	1.292	2.27E-03	1.299	0.246/0.201	3.42E-05	1.71E-04	1.305	0.224/0.220	0.929	1.009	0.773	1.294	0.029	3.70E-04	1.210
rs62533717	9	PTPRD	9.57E-03	1.459	2.11E-03	1.722	0.071/0.045	8.73E-05	1.09E-04	1.555	0.066/0.060	0.918	1.016	0.654	1.530	0.028	1.42E-03	1.334
***P*<0.01 in South females and *P*<0.05 in North females, but *P>0.05* in males**																		
rs10950722	7	FERD3L	1.07E-02	1.356	3.94E-03	1.312	0.165/0.131	7.12E-05	1.27E-04	1.340	0.148/0.161	0.648	0.951	0.710	1.409	0.010	2.50E-03	1.201
rs2226302	21	KCNE1	1.25E-02	0.823	2.78E-03	0.811	0.408/0.459	9.01E-05	9.79E-05	0.816	0.433/0.426	0.763	1.024	1.256	0.796	0.017	2.32E-03	0.876
rs6917116	6	PDE10A	2.22E-02	0.755	1.33E-03	0.685	0.089/0.118	6.68E-05	1.28E-04	0.713	0.123/0.118	0.725	1.044	1.463	0.684	0.010	9.77E-04	0.796
rs62461401	7	PCLO	2.27E-02	1.318	2.78E-04	1.501	0.138/0.102	2.58E-05	2.15E-04	1.412	0.123/0.131	0.673	0.950	0.673	1.486	0.007	1.54E-03	1.237
rs6468862	8	BAALC	2.70E-02	1.690	6.16E-04	1.821	0.046/0.027	4.12E-05	1.72E-04	1.779	0.039/0.037	0.774	1.060	0.596	1.678	0.037	2.94E-04	1.508

© 2018 Zeng Y et al. *JAMA Network Open.*

eTable 6. Power estimates for male- and female-specific GWAS

eTable 6a. Power estimates for the *male-specific GWAS* based on the CLHLS data: the minimum detectable odds ratios of the effects size of genotypes to reach a statistical power >80%, using QUANTO (1.1) software to perform the power calculation

Ideal p-value	$p<5\times10^{-8}$	$p<5\times10^{-7}$	$p<5\times10^{-6}$	$p<5\times10^{-5}$	$p<5\times10^{-4}$	$p<5\times10^{-3}$
Number of SNPs	0.82M geno-typed & 4.78M imputed SNPs	0.82M geno-typed & 4.78M imputed SNPs	0.82M geno-typed & 4.78M imputed SNPs	0.82M geno-typed & 4.78M imputed SNPs	0.82M geno-typed & 4.78M imputed SNPs	0.82M geno-typed & 4.78M imputed SNPs
Analysis	Association with longevity	Association with longevity	Association with longevity	Association with longevity	Association with longevity	Association with longevity
Sample size	564 centenarians & 773 controls	564 centenarians & 773 controls	564 centenarians & 773 controls	564 centenarians & 773 controls	564 centenarians & 773 controls	564 centenarians & 773 controls
Dep. var.	Reaching age 100+	Reaching age 100+	Reaching age 100+	Reaching age 100+	Reaching age 100+	Reaching age 100+
Prevalence	$2.33x10^{-6}$	$2.33x10^{-6}$	$2.33x10^{-6}$	$2.33x10^{-6}$	$2.33x10^{-6}$	$2.33x10^{-6}$
MAF=0.05	2.52	2.39	2.25	2.11	1.95	1.78
MAF=0.10	2.05	1.97	1.87	1.78	1.67	1.55
MAF=0.20	1.78	1.71	1.65	1.57	1.50	1.41
MAF=0.30	1.68	1.63	1.57	1.50	1.44	1.36
MAF=0.40	1.65	1.60	1.54	1.48	1.41	1.34

© 2018 Zeng Y et al. *JAMA Network Open.*

eTable 6b. Power estimates for the *female-specific* GWAS based on the CLHLS data: the minimum detectable odds ratios of the effects size of genotypes to reach a statistical power >80%, using QUANTO (1.1) software to perform the power calculation

Ideal p-value	$p<5\times10^{-8}$	$p<5\times10^{-7}$	$p<5\times10^{-6}$	$p<5\times10^{-5}$	$p<5\times10^{-4}$	$p<5\times10^{-3}$
Number of SNPs	0.82M geno-typed & 4.78M imputed SNPs	0.82M geno-typed & 4.78M imputed SNPs	0.82M geno-typed & 4.78M imputed SNPs	0.82M geno-typed & 4.78M imputed SNPs	0.82M geno-typed & 4.78M imputed SNPs	0.82M geno-typed & 4.78M imputed SNPs
Analysis	Association with longevity	Association with longevity	Association with longevity	Association with longevity	Association with longevity	Association with longevity
Sample size	1,614 centenarian & 1,526 controls	1,614 centenarian & 1,526 controls	1,614 centenarian & 1,526 controls	1,614 centenariar & 1,526 controls	1,614 centenarian & 1,526 controls	1,614 centenariar & 1,526 controls
Dep. var.	Reaching age 100+	Reaching age 100+	Reaching age 100+	Reaching age 100+	Reaching age 100+	Reaching age 100+
Prevalence	$7.83x10^{-6}$	$7.83x10^{-6}$	$7.83x10^{-6}$	$7.83x10^{-6}$	$7.83x10^{-6}$	$7.83x10^{-6}$
MAF=0.05	1.89	1.82	1.75	1.67	1.58	1.48
MAF=0.10	1.63	1.58	1.53	1.47	1.41	1.34
MAF=0.20	1.46	1.43	1.39	1.35	1.31	1.26
MAF=0.30	1.41	1.38	1.34	1.31	1.27	1.22
MAF=0.40	1.38	1.35	1.32	1.29	1.25	1.21

eTable 7. Parameters used and outcome of power estimates for the sex-specific PRS analyses on longevity based on CLHLS GWAS data, using the *AVENGEME* software [29]

	Males			Females		
	11 loci ($P<10^{-5}$)	51 loci ($P<10^{-4}$)	136 loci ($P<10^{-3}$)	12 loci ($P<10^{-5}$)	65 loci ($P<10^{-4}$)	290 loci ($P<10^{-3}$)
nsnp	394,403	394,403	394,403	394,403	394,403	394,403
discovery/ target	794/543	794/543	794/543	1733/1407	1733/1407	1733/1407
vg1	0.15	0.15	0.15	0.15	0.15	0.15
cov12	0.03610	0.07558	0.38726	0.03650	0.07808	0.38726
π0	0.99999	0.99991	0.99910	0.99999	0.99993	0.99896
Pupper	$0, 1x10^{-5}$	$0, 1x10^{-4}$	$0, 1x10^{-3}$	$0, 1x10^{-5}$	$0, 1x10^{-4}$	$0, 1x10^{-3}$
Prevalence	$2.33x10^{-6}$	$2.33x10^{-6}$	$2.33x10^{-6}$	$7.83x10^{-6}$	$7.83x10^{-6}$	$7.83x10^{-6}$
Sampling	0.3602	0.3602	0.3602	0.4784	0.4784	0.4784
Alpha	0.05	0.05	0.05	0.05	0.05	0.05
nested	True	True	True	True	True	True
weighted	True	True	True	True	True	True
binary	True	True	True	True	True	True
lambdaS	--	--	--	--	--	--
shrinkage	False	False	False	False	False	False
logrisk	False	False	False	False	False	False
Power	0.99747	0.99999	0.99915	1.00	1.00	1.00

Notes: The following definitions of the parameters were taken from the Users' Manual of the *AVENGEME* software [29]

1) nsnp: number of independent SNPs in the GWAS. Clumping was performed from the total 5,595,657 SNPs using PLINK to retain SNPs with R2<0.1 within 250 kb windows.
2) discovery/target: size of discovery sample / size of target sample.
3) vg1: proportion of trait variance explained by the entire set of SNPs in the discovery sample.
4) cov12: covariance between genetic effects in the discovery and target samples, estimated by the estimatePolygenic Model function in AVENGEME.

© 2018 Zeng Y et al. *JAMA Network Open.*

5) π0 = 1- (# of sex-specific SNPs ($P<10^{-5}$, 10^{-4}, or 10^{-3} in one sex but P>0.05 in other sex) among entire set of 5.6 million SNPs) / entire set of 5.6 million SNPs
6) pupper: lower bound, upper bound.
7) prevalence: Sex-specific prevalence rate of centenarians among the Chinese population.
8) sampling: (# of cases) / (# of total samples) in the discovery sample.
9) alpha: Default 0.05; no need to do Bonferroni correction since all selected SNPs are included in PRS and only one trait is considered [31].
10) nested: Only one interval.
11) weighted: PRS is constructed with weight *log(OR).*
12) binary: The trait "longevity" is binary.
13) shrinkage: Ridge regression model is not used.
14) logrisk: Liability scale is assumed.
15) The more detailed definitions of the parameters used for the power estimates of sex-specific PRS analyses are referred to in the manual of the R program for PRS power estimates *AVENGEME* [29,32]

© 2018 Zeng Y et al. *JAMA Network Open.*

Supplement eFigures

eFigure 1. Manhattan plots showing association with longevity: male

1a. *male discovery* (North region) GWAS dataset

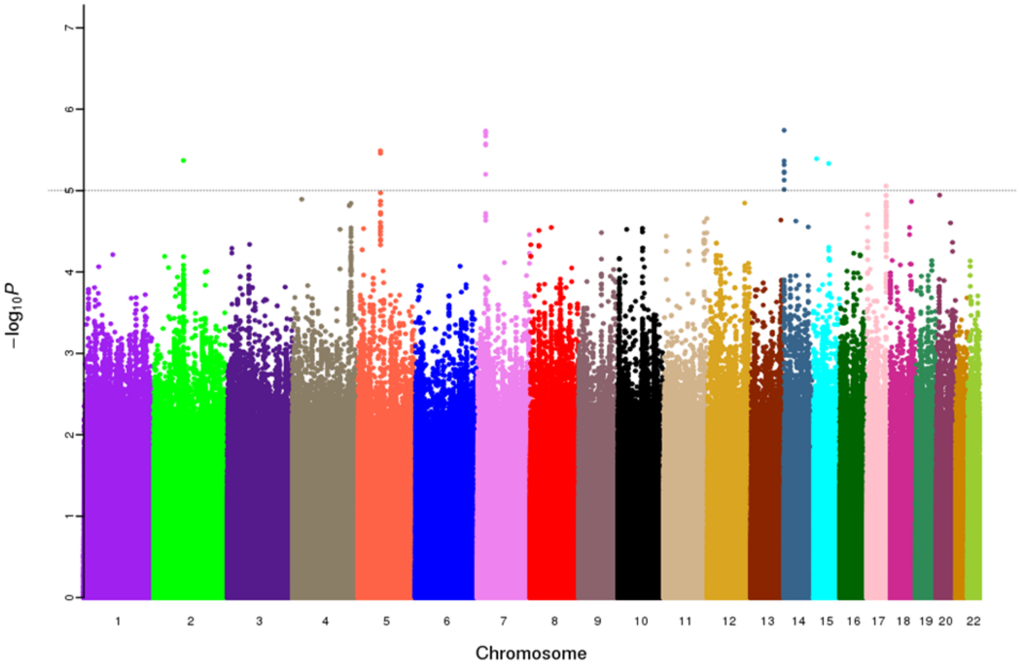

1b. *male evaluation* (South region) GWAS dataset

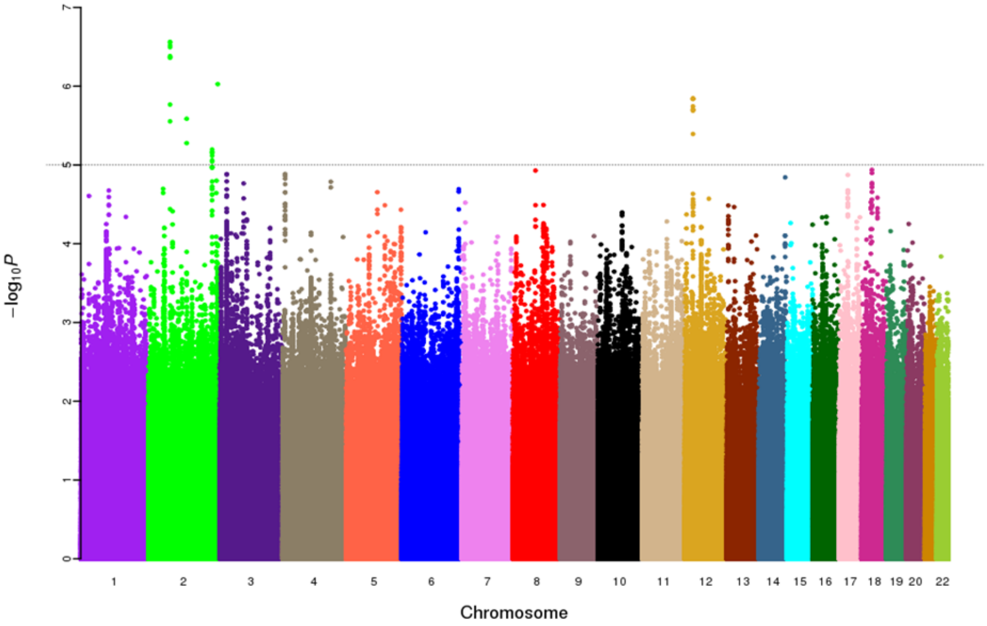

1c. *male North-South combined* GWAS dataset

© 2018 Zeng Y et al. *JAMA Network Open.*

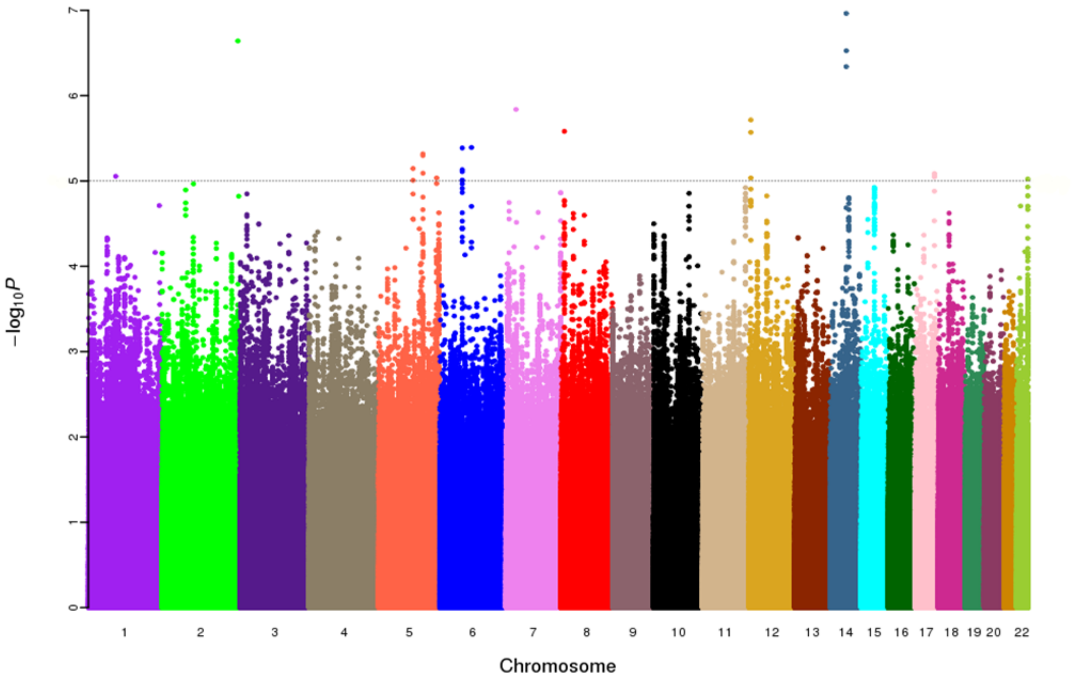

eFigure 2. Manhattan plots showing association with longevity: female

2a. *female discovery* (North region) GWAS dataset

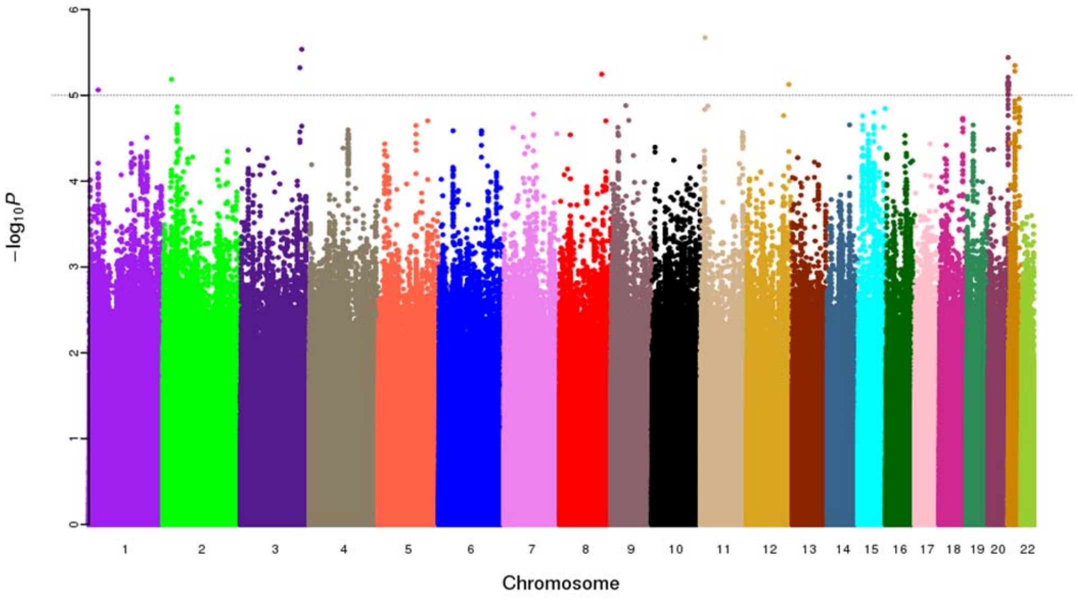

© 2018 Zeng Y et al. *JAMA Network Open.*

2b. *female evaluation* (South region) GWAS dataset

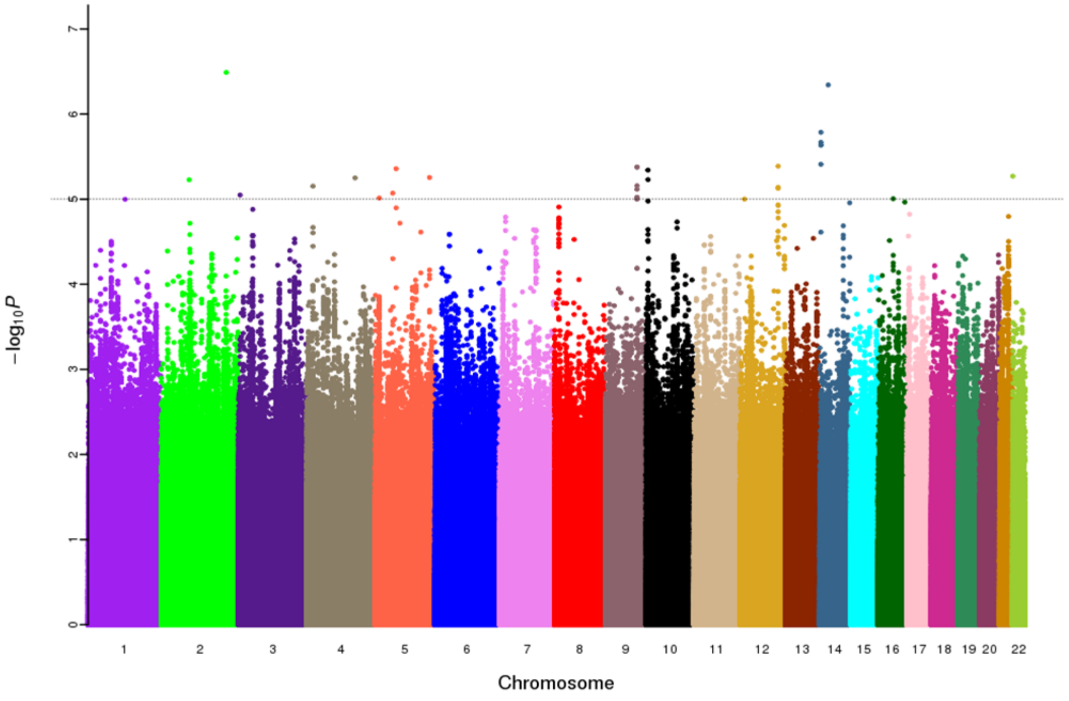

2c. *female North-South combined* GWAS dataset

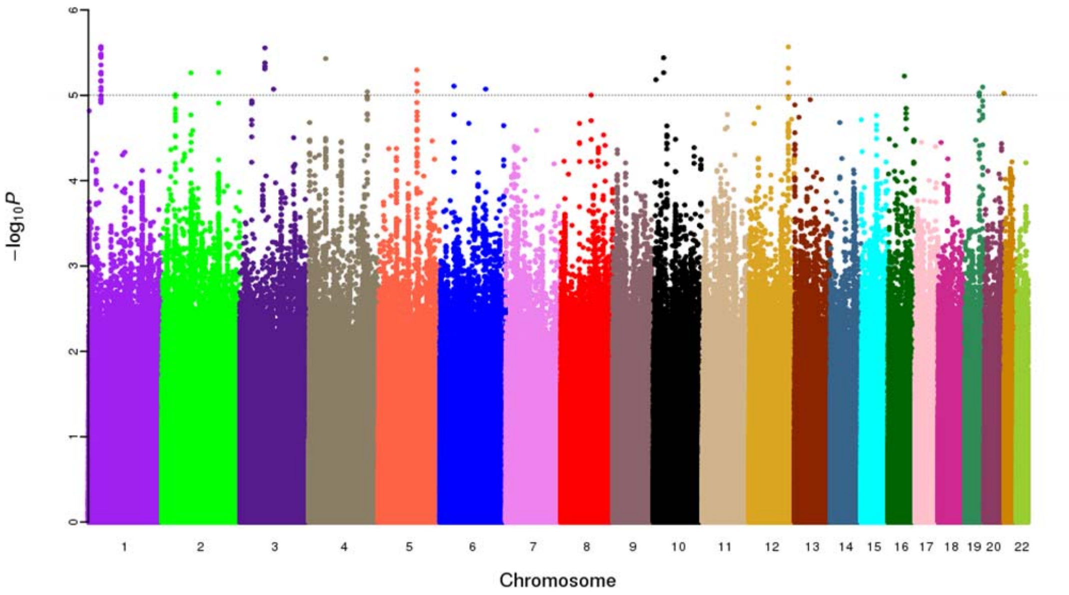

© 2018 Zeng Y et al. *JAMA Network Open.*

eFigure 3a. Quantile-quantile plots: male

3a. *male discovery* (North region) GWAS dataset (the genomic inflation factor is 1.020 (λ=1.020))

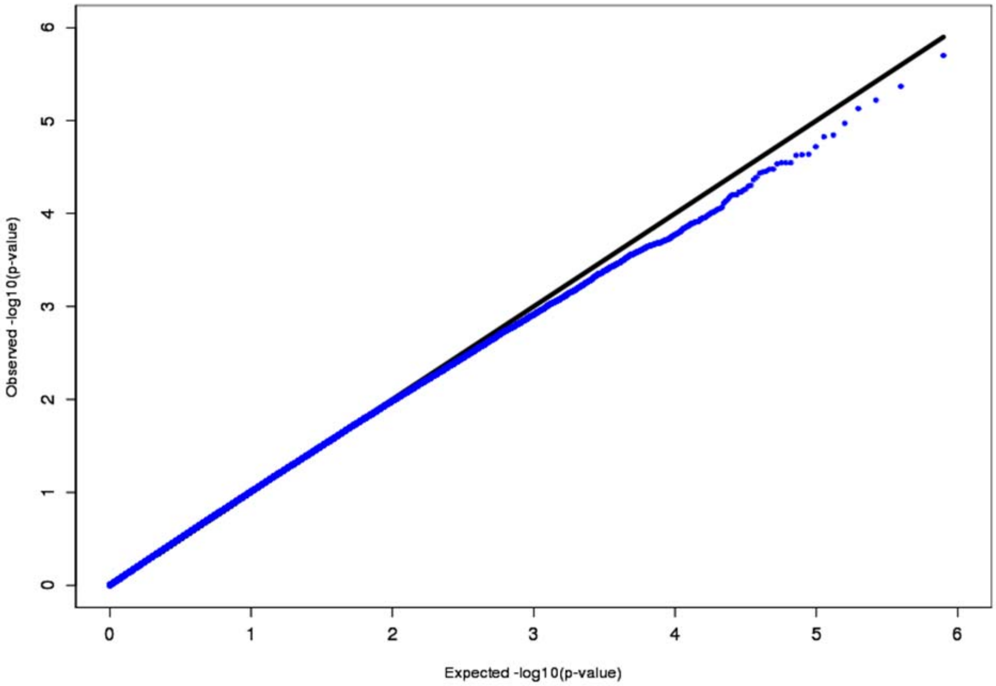

3b. *male evaluation* (South region) GWAS dataset (the genomic inflation factor is 1.010 (λ=1.010))

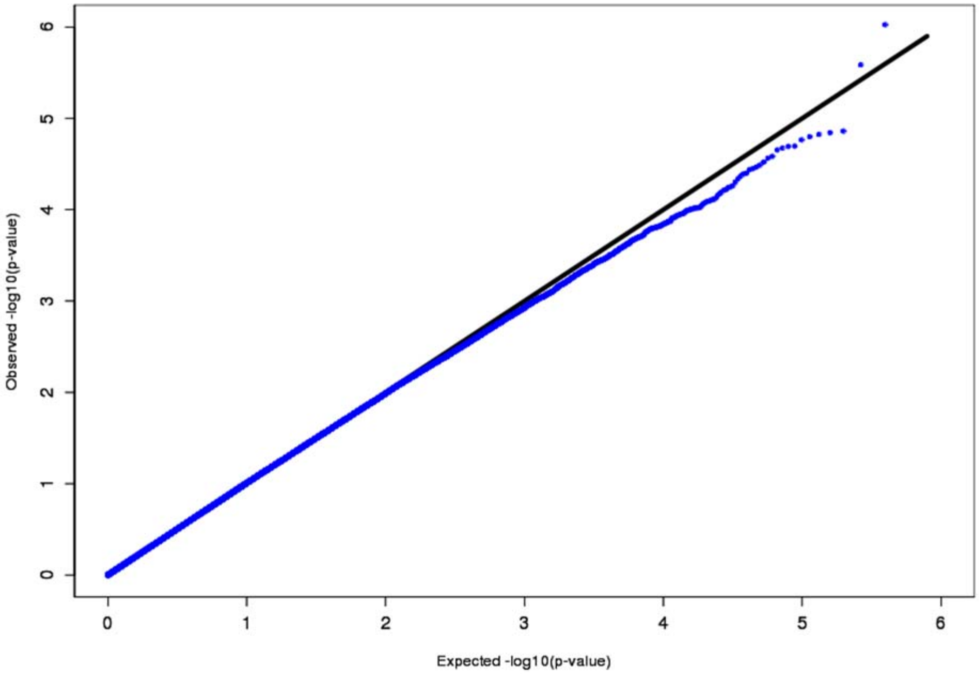

© 2018 Zeng Y et al. *JAMA Network Open.*

3c. *male North-South combined* GWAS dataset (the genomic inflation factor is 1.014 (λ=1.014))

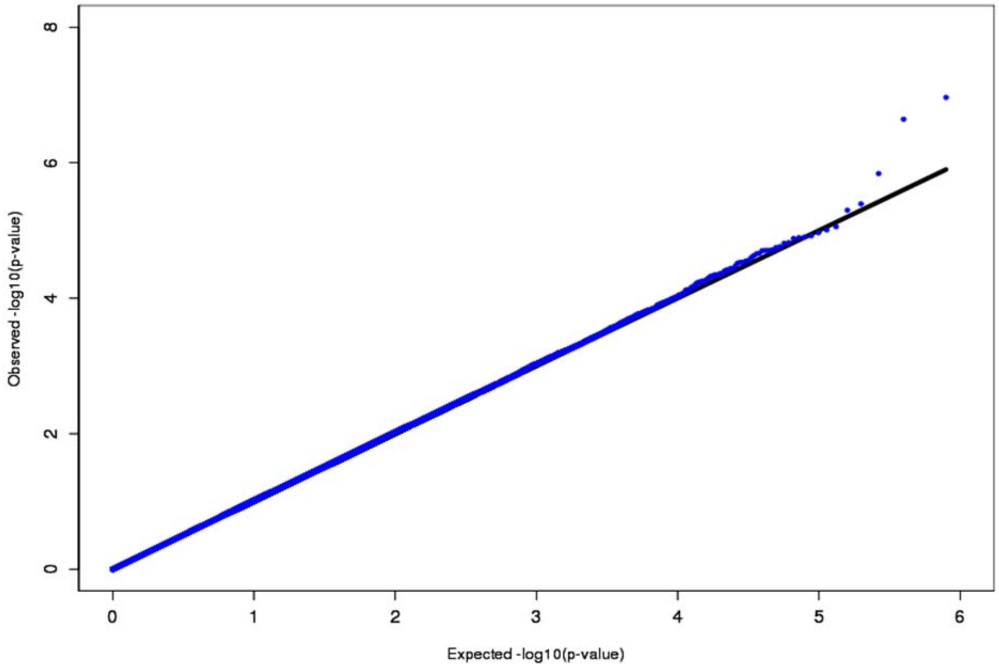

eFigure 4. Quantile-quantile plots: female

4a. *female discovery* (North region) GWAS dataset (the genomic inflation factor is 1.025 (λ=1.025)).

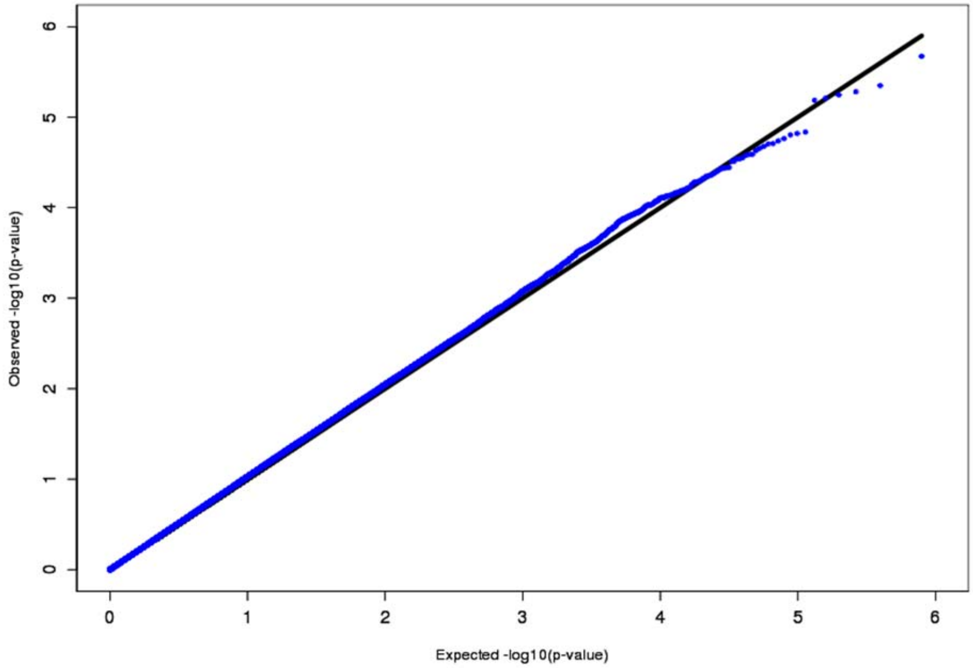

© 2018 Zeng Y et al. *JAMA Network Open.*

4b. *female evaluation* (South region) GWAS dataset (the genomic inflation factor is 1.016 (λ=1.016)).

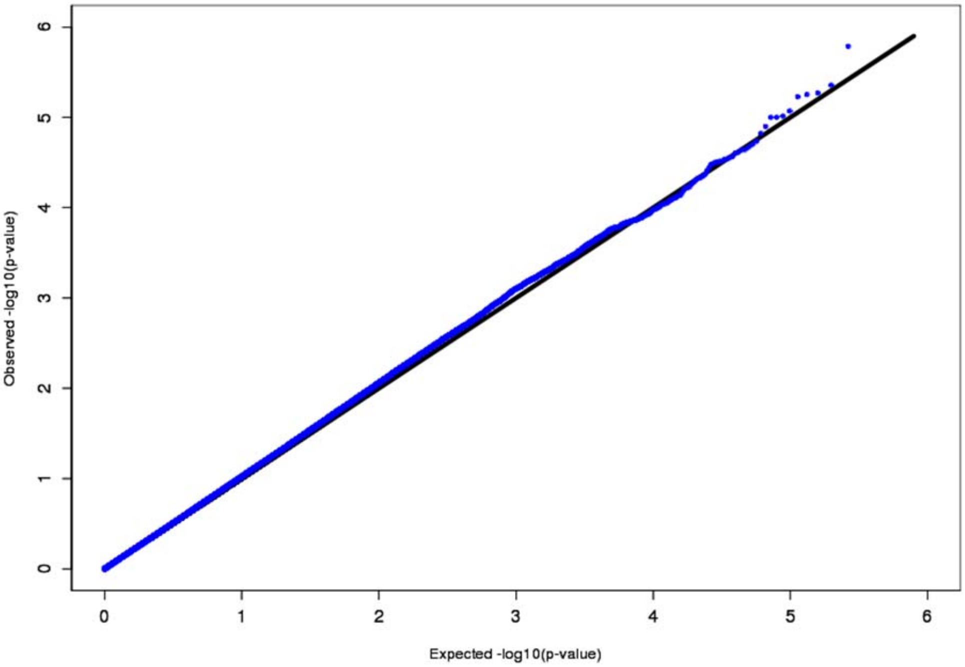

4c. *female North-South combined* GWAS dataset (the genomic inflation factor is 1.022 (λ=1.022)).

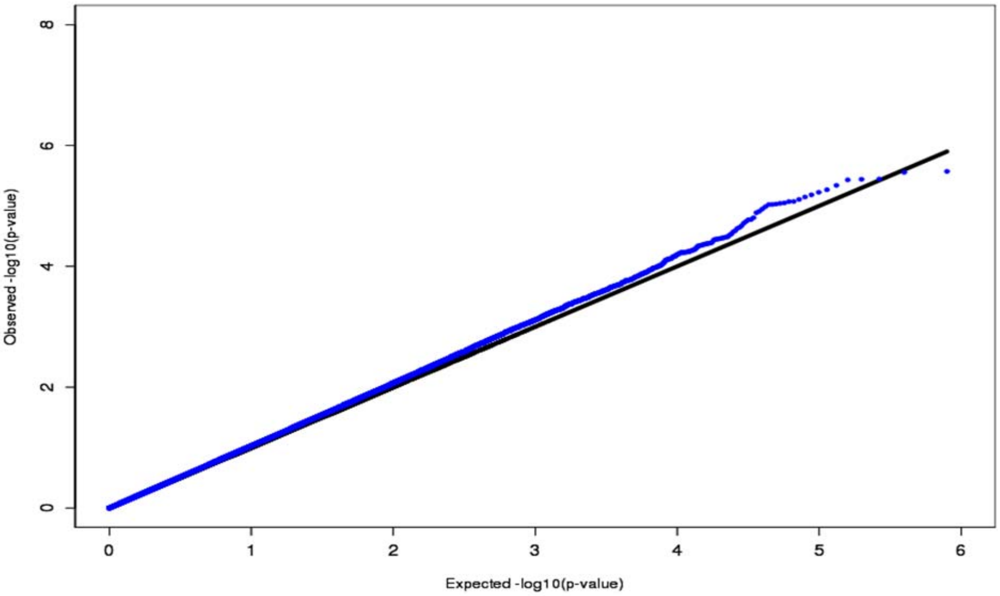

© 2018 Zeng Y et al. *JAMA Network Open.*
